# ATGL/CGI-58-Dependent Hydrolysis of a Lipid Storage Pool in Murine Enterocytes

**DOI:** 10.1016/j.celrep.2019.07.030

**Published:** 2019-08-13

**Authors:** Melanie Korbelius, Nemanja Vujic, Vinay Sachdev, Sascha Obrowsky, Silvia Rainer, Benjamin Gottschalk, Wolfgang F. Graier, Dagmar Kratky

**Affiliations:** 1 Gottfried Schatz Research Center, Molecular Biology and Biochemistry, Medical University of Graz, 8010 Graz, Styria, Austria; 2 BioTechMed-Graz, 8010 Graz, Styria, Austria

## Abstract

As circulating lipid levels are balanced by the rate of lipoprotein release and clearance from the plasma, lipid absorption in the small intestine critically contributes to the maintenance of whole-body lipid homeostasis. Within enterocytes, excessive triglycerides are transiently stored as cytosolic lipid droplets (cLDs), and their mobilization sustains lipid supply during interprandial periods. Using mice lacking adipose triglyceride lipase (ATGL) and its coactivator comparative gene identification-58 (CGI-58) exclusively in the intestine (intestine-specific double KO [iDKO]), we show that ATGL/CGI-58 are not involved in providing substrates for chylomicron synthesis. Massive intestinal cLD accumulation in iDKO mice independent of dietary lipids together with inefficient lipid incorporation into cLDs in the early absorption phase demonstrate the existence of a secretion/re-uptake cycle, corroborating the availability of two diverse cLD pools. This study identified ATGL/CGI-58 as critical players in the catabolism of basolaterally (blood) derived lipids and highlights the necessity to modify the current model of intestinal lipid metabolism.

## Introduction

Lipid metabolism comprises anabolic (lipogenic) and catabolic (lipolytic) processes, for which the small intestine (SI) plays a crucial role in maintaining systemic energy homeostasis. After food intake, dietary lipids such as triglycerides (TGs), cholesteryl esters (CEs), and phospholipids (PLs) are initially cleaved by oral and gastric hydrolases. The majority of luminal hydrolysis of TGs into free fatty acids (FFAs) and 2-monoacylglycerol is catalyzed by pancreatic lipase (PTL). These hydrolysis products are further emulsified with bile acids and packed into micelles in the lumen of the SI, where they are taken up by the apical side of enterocytes, either via passive diffusion or by protein-mediated transport mechanisms (Poirier et al., 1996; Phan and Tso, 2001; Abumrad and Davidson, 2012; Iqbal and Hussain, 2009). Absorbed TG precursors are re-esterified in the endoplasmic reticulum and either used for chylomicron (CM) synthesis or serve as a transient lipid storage pool in the form of cytosolic lipid droplets (cLDs). Together with very low density lipoproteins (VLDLs), CMs represent the main TG source for peripheral cells and tissues and are pivotal contributors to postprandial hypertriglyceridemia (Demignot et al., 2014). Therefore, the SI participates in the control of blood lipid concentrations and the development of cardiovascular diseases (Simons et al., 1987).

Generally, cLDs are essential organelles for the prevention of lipotoxicity by re-esterification of FFAs into TGs (Schaffer, 2003). cLDs also serve as a reservoir for hydrophobic molecules with important biological functions including fat-soluble vitamins, prostaglandins, and steroids (Demignot et al., 2014). The current hypothesis states that intestinal cLDs optimize lipid absorption during food intake and provide sustained lipid supply during fasting (Beilstein et al., 2016). The gut is also able to take up FFAs from the circulation, derived from white adipose tissue (WAT) or vascular lipolysis, hepatic-derived small lipoprotein particles, or CM remnants (Soued and Mansbach, 1996).

Recent findings indicate the existence of two different TG pools in enterocytes, as evidenced by diverging fates of apically (diet) and basolaterally (circulation) derived lipids. Although lipids taken up via the apical brush border membrane are mainly incorporated into TGs and directed toward the secretory pathway, basolateral lipids are destined for fatty acid (FA) β-oxidation or phospholipid (PL) synthesis (Storch et al., 2008; Ho et al., 2002; Gangl and Ockner, 1975). This might be due to different compartmentation of intracellular organelles, as mitochondria are dispersed throughout the enterocyte but predominate at the basal pole, while the endoplasmic reticulum is concentrated rather on the apical side of the cell (Gangl and Ockner, 1975). In humans, sequential meal studies described the existence of transient intestinal lipid storage pools, as TGs stored in cLDs showed the same FA composition as TGs ingested with the previous meal (Fielding et al., 1996; Evans et al., 1998; Jackson et al., 2002). In mice, the number and size of cLDs increase during dietary fat absorption with a maximum 3 h post-gavage and an almost complete depletion 12 h after digestion (Zhu et al., 2009). Lipolysis is necessary to mobilize stored lipids, which are further used for energy production and membrane biosynthesis (Farese and Walther, 2009) or serve as ligands for transcription factors (Zechner et al., 2012). In WAT, lipolysis of TGs stored in cLDs has already been well described, and adipose TG lipase (ATGL) together with its coactivator comparative gene identification-58 (CGI-58) mediates the initial step of TG hydrolysis (Zimmermann et al., 2004; Yen and Farese, 2006; Lass et al., 2006). However, the mechanisms accountable for the breakdown of cLDs in the SI are still elusive.

We hypothesized that the enzymatic pathway responsible for the degradation of intestinal cLDs involves ATGL and CGI-58. Our previous study suggested a role of ATGL in the hydrolysis of a distinct intestinal lipid pool, as intestine-specific deletion of ATGL led to increased cLD accumulation but unchanged TG absorption (Obrowsky et al., 2013), indicating that intestinal ATGL hydrolyzes TGs stored in cLDs but is dispensable for CM production. Lack of intestinal CGI-58 in mice also leads to increased cLD accumulation in murine enterocytes, even during the fasting state. Decreased postprandial plasma TG levels implicated that CGI-58 is required for efficient CM secretion in an ATGL-independent manner (Xie et al., 2014). Because of these diverging results with respect to the role of neutral lipolysis in lipid metabolism, we generated and characterized a mouse model lacking both ATGL and CGI-58 exclusively in the SI.

## Results

### Increased Intestinal Lipid Accumulation in iDKO Mice

**Figure 1:**
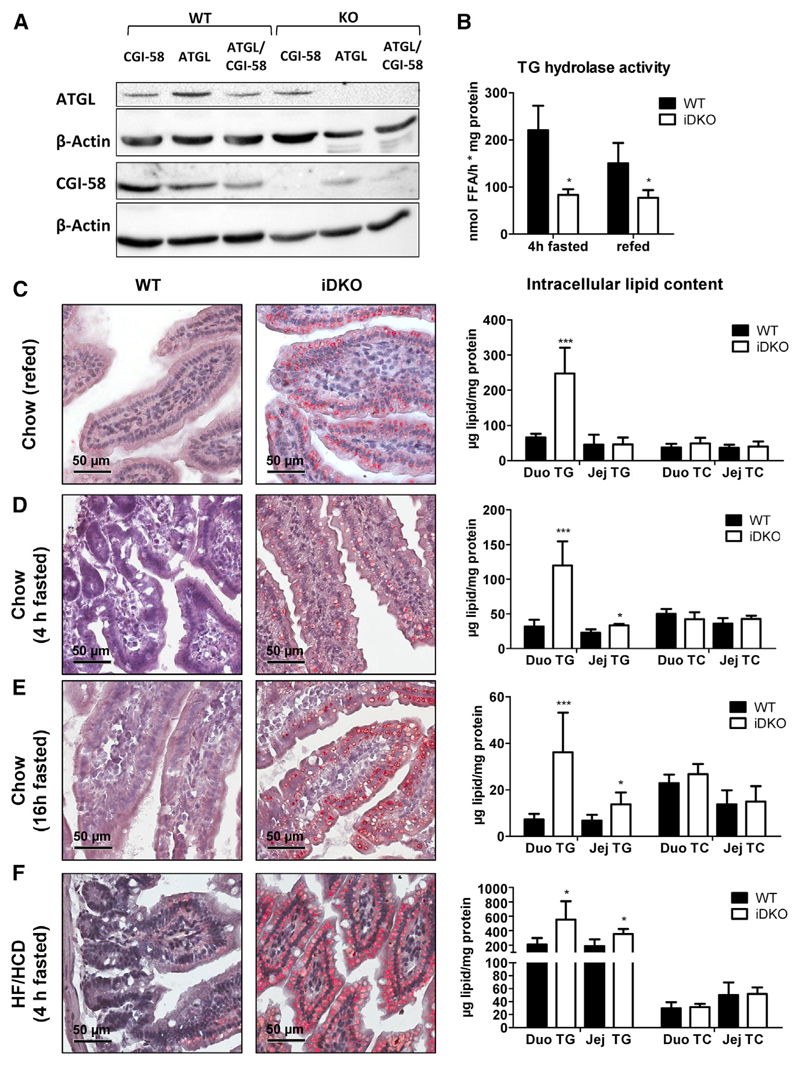
Loss of Intestinal ATGL and CGI-58 Leads to Augmented Intracellular Lipid Accumulation (A) Protein lysates (80 μg) were separated using SDS-PAGE, and protein expression levels of ATGL and CGI-58 in the jejunum of 4 h fasted, chow dietfed Atgl iKO, Cgi-58 iKO, and iDKO mice were determined using western blotting. Monoclonal anti-mouse β-actin served as loading control. (B) TG hydrolase activity in the jejunum of chow diet-fed iDKO mice in 4 h fasted (n = 3 or 4) and refed states (12 h fasting, 2 h refeeding; n = 4 or 5). (C–F) ORO staining of duodenal cryosections and biochemical quantification of intracellular lipid concentrations in chow diet-fed iDKO mice after refeeding (n = 4 or 5; C), fasted for 4 h (n = 3 or 4; D) or 16 h (n = 6–9; E), and in mice challenged with HF/HCD for 5 weeks and fasted for 4 h (n = 4 or 5; F). Data represent mean + SD. *p < 0.05 and ***p ≤ 0.001. Magnification, 40×; scale bar, 50 μm. Duo, duodenum; Jej, jejunum; TC, total cholesterol; TG, triglycerides. See also Figure S1.

To investigate the role of intestinal ATGL and CGI-58 in systemic lipid homeostasis, we generated mice specifically lacking ATGL (Atgl intestine-specific KO [iKO]), CGI-58 (Cgi-58 iKO), or both proteins (intestine-specific double KO [iDKO]) in enterocytes. Protein expression of ATGL and/or CGI-58 was absent in the SI of mice harboring a single or double deficiency of ATGL or CGI-58, respectively (Figure 1A). iDKO mice and mice carrying a single deletion of ATGL or CGI-58 showed a marked reduction in *Atgl* (Figures S1A and S1C) and *Cgi-58* (Figures S1B and S1D) mRNA expression along the SI, without affecting hepatic expression of either gene. Decreased neutral TG hydrolase activity in the jejunum of chow diet-fed iDKO mice independent of the feeding state (Figure 1B) strongly suggested critical roles for intestinal ATGL and CGI-58 in gut lipolysis.

Under various dietary conditions and feeding states, iDKO and control mice had comparable body weight (Table 1), food intake, fecal output, and fecal lipid composition (Figures S1E–S1G). Oil red O (ORO) staining of duodenal sections from chow diet-fed (Figures 1C–1E) and high-fat, high-cholesterol diet (HF/HCD)-fed (Figure 1F) iDKO mice revealed increased lipid deposition exclusively in enterocytes. In the refed state, we observed a 3.8-fold increase in intestinal TG levels solely in the duodenum (Figure 1C). Moreover, duodenal and jejunal TG concentrations of chow diet-fed iDKO mice fasted for 4 h were elevated by 3.8- and 1.5-fold, respectively (Figure 1D). Even 16 h fasted iDKO mice showed persistent cLD accumulation and increased duodenal (4.4-fold) and jejunal (2.0-fold) TG levels compared with wild-type (WT) mice (Figure 1E). Challenging iDKO mice with HF/HCD for 5 weeks resulted in increased TG concentrations in the duodenum (2.6-fold) and jejunum (1.9-fold) (Figure 1F).

### Luminal Lipid Absorption Is Unaffected by Intestinal ATGL/CGI-58 Deficiency

**Figure 2:**
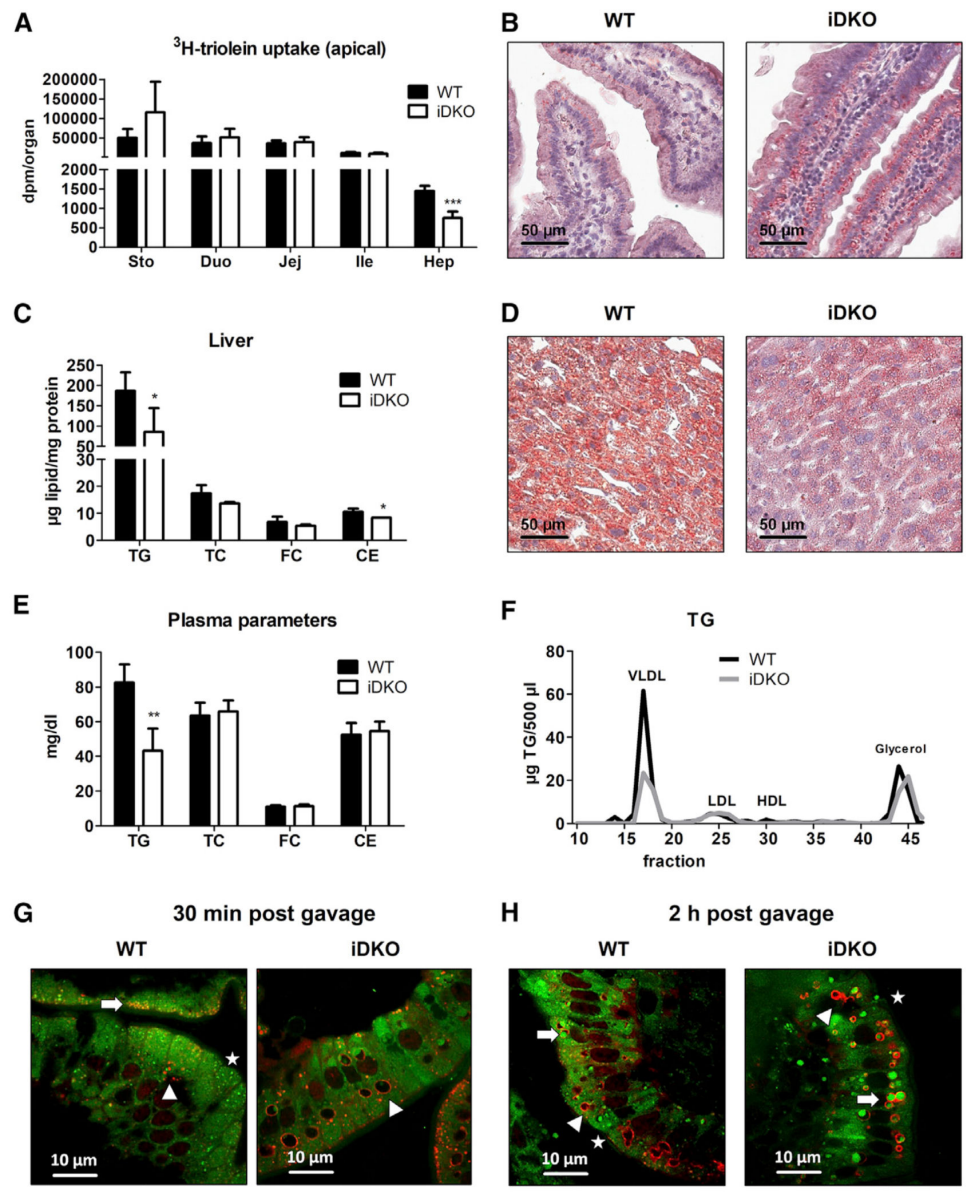
Intestinal Loss of ATGL and CGI-58 Ameliorates Hepatic Steatosis 30 min Post-gavage (A) Radioactivity in the SI and liver of chow diet-fed WT and iDKO mice (n = 4 or 5) 30 min post-gavage of 2 μCi [9,10-^3^H(N)]-triolein in 200 μL corn oil. (B) ORO staining of duodenal sections 30 min after an oral lipid load. (C and D) Biochemical (C) and histological (D) analysis of hepatic lipid levels 30 min post-gavage of 200 μL corn oil. (E and F) Lipid concentrations (E) and (F) lipoprotein profiles in the plasma 30 min post-gavage of corn oil (200 μL). Data represent mean + SD (n = 3 or 4). *p < 0.05, **p ≤ 0.01, and ***p ≤ 0.001. Magnification, 40×; scale bar, 50 μm. (G and H) Chow diet-fed mice were fasted for 16 h prior to an oral administration of 100 μL corn oil containing 1 μg/g body weight BODIPY FL C_16_ (green). Intestinal sections were co-stained with PLIN3 (red) to visualize colocalization with cLDs. PLIN3 immunofluorescence staining 30 min (G) and 2 h (H) post-gavage. Green background fluorescence results from high chlorophyll content in the chow diet (alfalfa). Arrows indicate cLDs originating from BODIPY-labeled FA, which colocalize with PLIN3; arrowheads indicate endogenous cLDs coated with PLIN3; stars indicate BODIPY-containing cLDs, which do not colocalize with PLIN3. Magnification, 100×; scale bar, 10 μm. CE, cholesteryl esters; Duo, duodenum; FC, free cholesterol; HDL, high-density lipoprotein; Hep, hepar (liver); Ile, ileum; Jej, jejunum; LDL, low-density lipoprotein; PLIN3, Perilipin 3; Sto, stomach; TC, total cholesterol; TG, triglycerides; VLDL, very low density lipoprotein. See also Figure S2.

To elucidate whether lipids accumulating to a higher extent in the SI of iDKO mice derive from the diet, mice were sacrificed 30 min post-gavage of corn oil containing ^3^H-triolein, mimicking the early absorption phase. WT and iDKO mice displayed comparable radioactivity in the SI (Figure 2A) but an increased number of cLDs in the duodenum (Figure 2B), indicating rather preformed cLDs than newly absorbed lipids from the gavage. Interestingly, we observed a 48% decrease of radioactivity in the liver of iDKO mice (Figure 2A) due to 45% and 20% reductions in hepatic TG and CE concentrations, respectively (Figure 2C). Histological analysis confirmed the ameliorative effect of intestinal ATGL/CGI-58 deficiency on hepatic steatosis (Figure 2D), which might be attributable to 48% decreased plasma TG levels in the VLDL fraction (Figures 2E and 2F).

Because the SI of iDKO and WT mice accumulated comparable amounts of dietary lipids in the early absorption phase, we next traced the fate and time course of TGs within enterocytes by gavaging mice with BODIPY FL C_16_, a fluorescently labeled FA analog. We chose this substrate because its FA chain length is likely sufficient to prevent its passive diffusion through the enterocyte into the circulation but rather requires re-esterification and thus allows visualizing TGs within enterocytes (Wang et al., 2013). Animals were sacrificed 30 min and 2 h post-gavage as described (Vasquez et al., 2011) to follow the fate of alimentary FAs *in vivo*. As perilipin 3 (PLIN3) protein expression on intestinal cLDs is upregulated in the early phase of dietary lipid absorption (Lee et al., 2009), we investigated PLIN3 expression after BODIPY FL C_16_ administration. After 30 min, we observed PLIN3 localization at the apical border of the enterocytes in WT mice (Figures 2G and S2A), reflecting the uptake and early formation of small cLDs containing BODIPY-labeled FAs. Mice lacking ATGL and CGI-58 mainly displayed increased accumulation of large cLDs containing endogenous lipids, detected as PLIN3-coated vesicles lacking BODIPY staining (Figures 2G and S2A), indicating that 30 min is not sufficient for dietary lipids to accumulate in the SI of iDKO mice. Two hours post-gavage, however, iDKO mice showed an increased number of larger cLDs with prominent colocalization of PLIN3 compared with controls (Figures 2H and S2B), raising the idea of a basolateral re-uptake of lipids. Quantification of the cLD types (BODIPY-labeled cLDs, PLIN3-coated vesicles, PLIN3-coated BODIPY-containing cLDs) revealed a larger number of cLDs in iDKO mice (Figure S2C), which predominantly corresponded to BODIPY-containing cLDs colocalizing with PLIN3 (Figure S2D). BODIPY-containing cLDs without PLIN3 (20% of total cLDs in iDKO mice) might reflect earlier formed cLDs, which are instead coated with PLIN2.

### Intestinal ATGL and CGI-58 Do Not Provide Substrates for CM Synthesis

**Figure 3:**
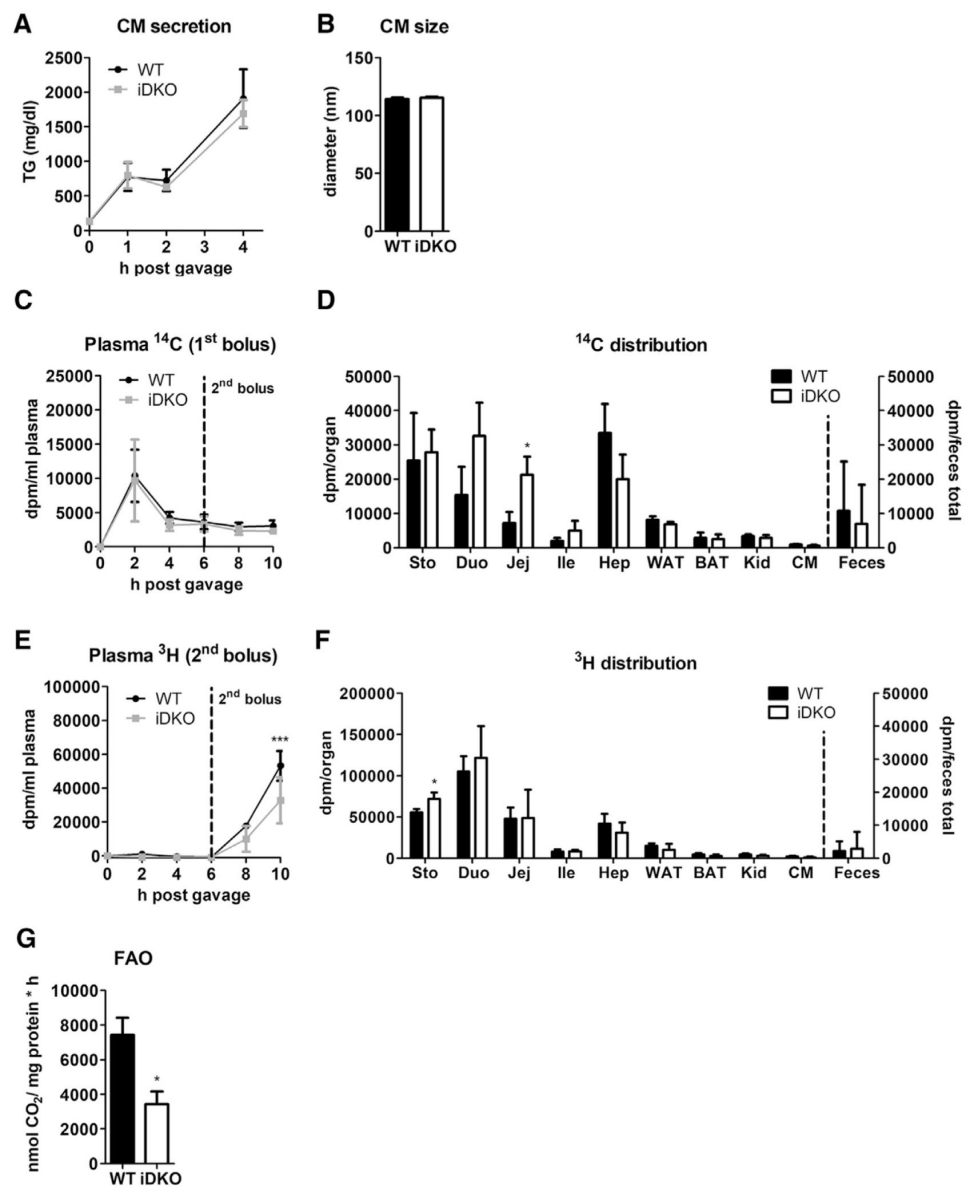
Atgl/Cgi-58 iDKO Mice Accumulate Lipids Ingested with the First Meal to a Greater Extent Than WT Mice (A) Thirty minutes after tyloxapol injection, 16 h fasted mice (n = 3–6) were gavaged with 200 μL olive oil. TG concentrations (resembling CM secretion) were determined 1, 2, and 4 h post-gavage. (B) Mice (n = 3) were fasted 4 h prior to injection of tyloxapol (500 mg/kg body weight). One hour later, mice received an oral olive oil bolus of 200 μL, and blood was drawn 90 min post-gavage. CM size was measured by light scattering. (C–F) After a 12 h fasting period, chow diet-fed mice (n = 3) were gavaged with 200 μL olive oil containing 0.5 μCi [1-^14^C]-triolein. Six hours after the first bolus, mice were gavaged with 200 μL olive oil containing 2 μCi [9,10-^3^H(N)]-triolein. Ten hours after the first gavage, mice were sacrificed, and organs were collected. (C) Secretion of ^14^C-labeled lipids from the initial meal into the circulation. (D) ^14^C distribution in tissues and feces reflects primarily ingested lipids. (E) ^3^H-labeled lipid secretion of the second meal administered at time point 6 h. (F) ^3^H-labeled lipid distribution in tissues and feces from the second meal. (G) Release of ^14^CO_2_ after incubation of isolated enterocytes (n = 3) with 0.4 mCi [1-^14^C]-palmitic acid. Data represent mean ± SD. *p < 0.05 and ***p ≤ 0.001. BAT, brown adipose tissue; CM, cardiac muscle (heart); Duo, duodenum; Hep, hepar (liver); Ile, ileum; Jej, jejunum; Sto, stomach; WAT, white adipose tissue. See also Figures S3 and S4.

With the exception of 32% decreased TG concentrations in iDKO mice fasted for 16 h, plasma lipid parameters were comparable between the genotypes mice under various dietary conditions (Table 1). These results indicate that ATGL/CGI-58 are not involved in the hydrolysis of dietary lipids to provide FFAs as substrates for CM synthesis. To corroborate this finding, we injected mice with tyloxapol to inhibit peripheral lipolysis, followed by an oral oil bolus. Identical CM secretion capacities (Figure 3A) and unaffected CM size (Figure 3B) strongly suggested that ATGL and CGI-58 lack essential roles in intestinal lipoprotein metabolism. Although intestinal CGI-58 was suggested to affect circulating postprandial TG levels (Xie et al., 2014), HF/HCD-fed Cgi-58 iKO mice showed identical TG secretion rates after an oral lipid bolus (Figure S3A). Accordingly, intestinal as well as hepatic mRNA expression of *Mttp* (Figures S3B and S3C), the rate-limiting enzyme for lipoprotein assembly in secretory tissues, and expression of the TG-synthesizing enzymes *Dgat1* and *Dgat2* (data not shown) remained comparable between WT and iDKO mice. Similar dietary lipid uptake in the early absorption phase (Figure 2A) together with unaltered secretion of dietaryderived TGs (Figure 3A) and fecal lipid loss (Figure S1G), however, indicates that apical lipid uptake into the enterocytes per se is not affected by the loss of intestinal ATGL and CGI-58.

### TG Hydrolysis by Intestinal ATGL and CGI-58 Does Not Provide Substrates for CMs Secreted in a Sequential Meal but Rather Serves for Enterocyte FA β-Oxidation

In humans, lipids ingested with a meal appear in the plasma after ingestion of a sequential meal (Fielding et al., 1996; Evans et al., 1998), emphasizing the existence of a transient intestinal lipid pool consisting of dietary lipids. To analyze this sequential meal effect in mice, we challenged iDKO and control mice with two differently labeled meals. Over a time period of 10 h, intestinal loss of ATGL and CGI-58 did not affect the secretion of the first substrate (^14^C-triolein) into the circulation (Figure 3C). Gavage of the second substrate (^3^H-triolein) 6 h later failed to trigger the secretion of previously ingested lipids in WT mice (Figure 3C), suggesting that intestinal fat turnover occurs differently in mice and humans. Despite comparable circulating ^14^C levels, representing the primary oil bolus, ^14^C counts were increased in the proximal parts of the SI in iDKO mice 10 h post-gavage (Figure 3D), but remained comparable between the genotypes in other tissues, such as liver, white and brown adipose tissue, kidney, heart, and feces. However, iDKO mice secreted 38% less lipids administered with the second gavage 10 h post-ingestion of the primary meal (Figure 3E). Except in the stomach, ^3^H-labeled lipids ingested with the second oil bolus failed to accumulate to a higher extent in the tissues of iDKO mice (Figure 3F). Fecal excretion (Figure 3F) and organ weights (Figure S4A) remained comparable between the genotypes.

We have previously shown that FFAs released by ATGL-mediated TG hydrolysis in enterocytes function as signaling molecules and participate in PPARα activation within enterocytes (Obrowsky et al., 2013). However, increased accumulation of cLDs in iDKO mice solely at later time points of dietary lipid absorption raised the idea of a non-alimentary source of lipids. Although lipids deriving from the basolateral side of the enterocyte may be destined for PL synthesis (Storch et al., 2008; Ho et al., 2002; Gangl and Ockner, 1975), incorporation of both ^3^H and ^14^C into the PL fraction remained comparable in iDKO mice (Figures S4B and S4C). This result indicates that products generated by intestinal ATGL and CGI-58 are unlikely shuttled into the PL synthesis pathway. Besides PL synthesis, basolaterally derived FAs can also be used for energy production (Storch et al., 2008; Ho et al., 2002; Gangl and Ockner, 1975). We therefore isolated jejunal enterocytes from WT and iDKO mice and incubated them with ^14^C-palmitic acid. ^14^CO_2_ trapped for 2 h revealed a 54% decrease in intestinal FA oxidation rate (Figure 3G), suggesting that intestinal neutral lipolysis shuttles FFAs to mitochondria as energy substrate.

### Intestinal ATGL and CGI-58 Hydrolyze Lipids Derived from the Basolateral Side

**Figure 4:**
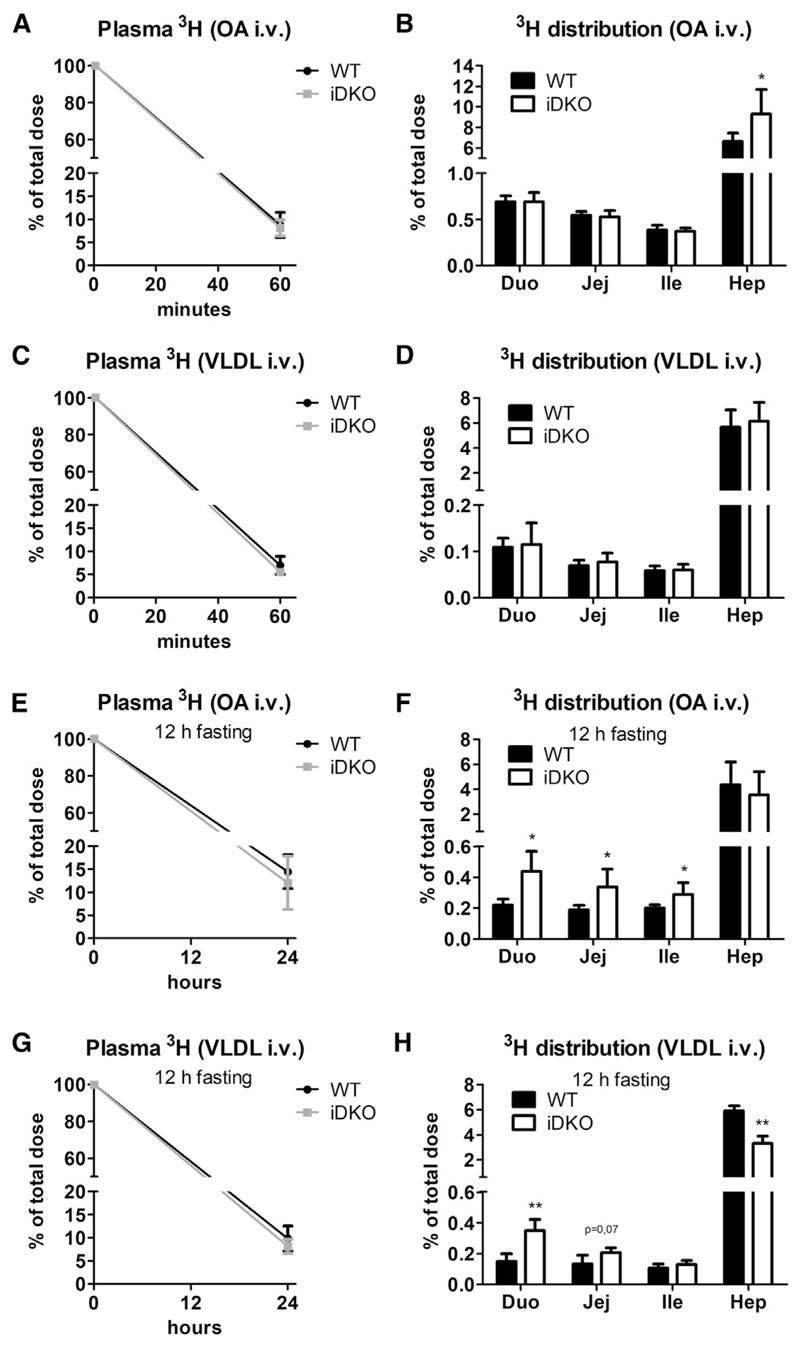
ATGL/CGI-58 Deficiency Causes Increased Accumulation of Basolaterally Absorbed VLDL Particles in the SI Mice were injected intravenously (i.v.) with 5 μCi [9,10-^3^H(N)]-oleic acid (OA) complexed with BSA or 1 μCi [9,10-^3^H(N)]-triolein incorporated into VLDL (2.44 mg TG). (A and B) Radioactivity in (A) plasma and (B) tissues 1 h after injection of ^3^H-OA (n = 5 or 6). (C and D) Radioactivity in (C) plasma and (D) tissues 1 h after injection of ^3^H-VLDL (n = 5). (E–H) Mice were sacrificed 24 h post-injection of the radioactive tracer, after a 12 h fasting period to trigger VLDL secretion. ^3^H-OA in plasma (E) and tissues (F) 24 h post-injection (n = 5–7). VLDL-TG-derived ^3^H levels in plasma (G) and tissues (H) (n = 3 or 4). Data represent mean + SD. *p < 0.05 and **p ≤ 0.01. Duo, duodenum; Hep, hepar (liver); Ile, ileum; Jej, jejunum. See also Figure S5.

To further assess whether basolaterally derived lipids are the source of TGs accumulating to a greater extent in the SI of iDKO mice, we injected mice intravenously (i.v.) either with FAs or TGs using ^3^H-oleic acid (OA) complexed with BSA or ^3^H-triolein incorporated into human VLDL. We observed no differences between the genotypes in radioactive counts disappearing from the circulation 1 h after injection (Figures 4A and 4C), and neither ^3^H-OA nor lipoprotein-derived ^3^H accumulated differently in the SI of iDKO and WT mice (Figures 4B and 4D). However, both radioactive tracers were mainly targeted to the liver (6%–9% of total dose), shuttling <1% to the enterocytes. These results were reconfirmed by i.v. injection of a fluorescently labeled FA (BODIPY FL C_16_), with substantial deposition in livers but less abundance in intestinal sections 30 min post-injection, irrespective of the genotype (Figures S5A and S5B). Co-staining with PLIN3 corroborated the accumulation of large cLDs containing endogenous lipids, detected as PLIN3-coated vesicles lacking BODIPY staining (Figure S5B), concordant with results observed 30 min post-gavage (Figure 2G).

Deposition of the tracer primarily in the liver indicated that lipids likely require processing by hepatocytes before redistribution to the SI. Therefore, we injected mice i.v. either with ^3^H-OA complexed to BSA or with human VLDL containing ^3^H-triolein. As neither 30 min (Figure S5B) nor 1 h (Figures 4B and 4D) was sufficient for both substrates to get incorporated into intestinal cLDs in iDKO mice, we sacrificed mice 24 h after injection, with a 12 h fasting period prior to sacrifice. This fasting period should trigger hepatic VLDL release and hence shed light on the contribution of hepatic lipoprotein metabolism on intestinal lipid homeostasis. Circulating radioactivity remained comparable between the genotypes in both experiments (Figures 4E and 4G). Radioactive counts deriving from both ^3^H-OA and ^3^H-VLDL accumulated to a higher extent in the proximal SI of iDKO mice (Figures 4F and 4H). ^3^H counts originating from ^3^H-OA were increased 2.0- and 1.8-fold in the duodenum and jejunum of iDKO mice, respectively (Figure 4F), while ^3^H levels applied via VLDL were 2.3-fold increased in the duodenum of iDKO mice, accompanied by a 44% reduction of hepatic counts (Figure 4H). However, the role of hepatocytes in processing of the substrate still remained elusive. Lipoprotein profiling of plasma samples 24 h post-injection revealed similar ^3^H distribution between the genotypes with the vast majority of the tracer present as albumin-bound FAs (Figure S5C). Although these findings argued against a critical role of hepatocytes and hepatic lipoprotein metabolism in sustaining lipid supply for the SI, results from these experiments corroborated the essential contribution of intestinal ATGL/CGI-58 in the catabolism of a basolaterally derived lipid pool.

### Atgl/Cgi-58 iDKO Mice Accumulate cLDs Even upon Restriction to Endogenous Lipids

**Figure 5:**
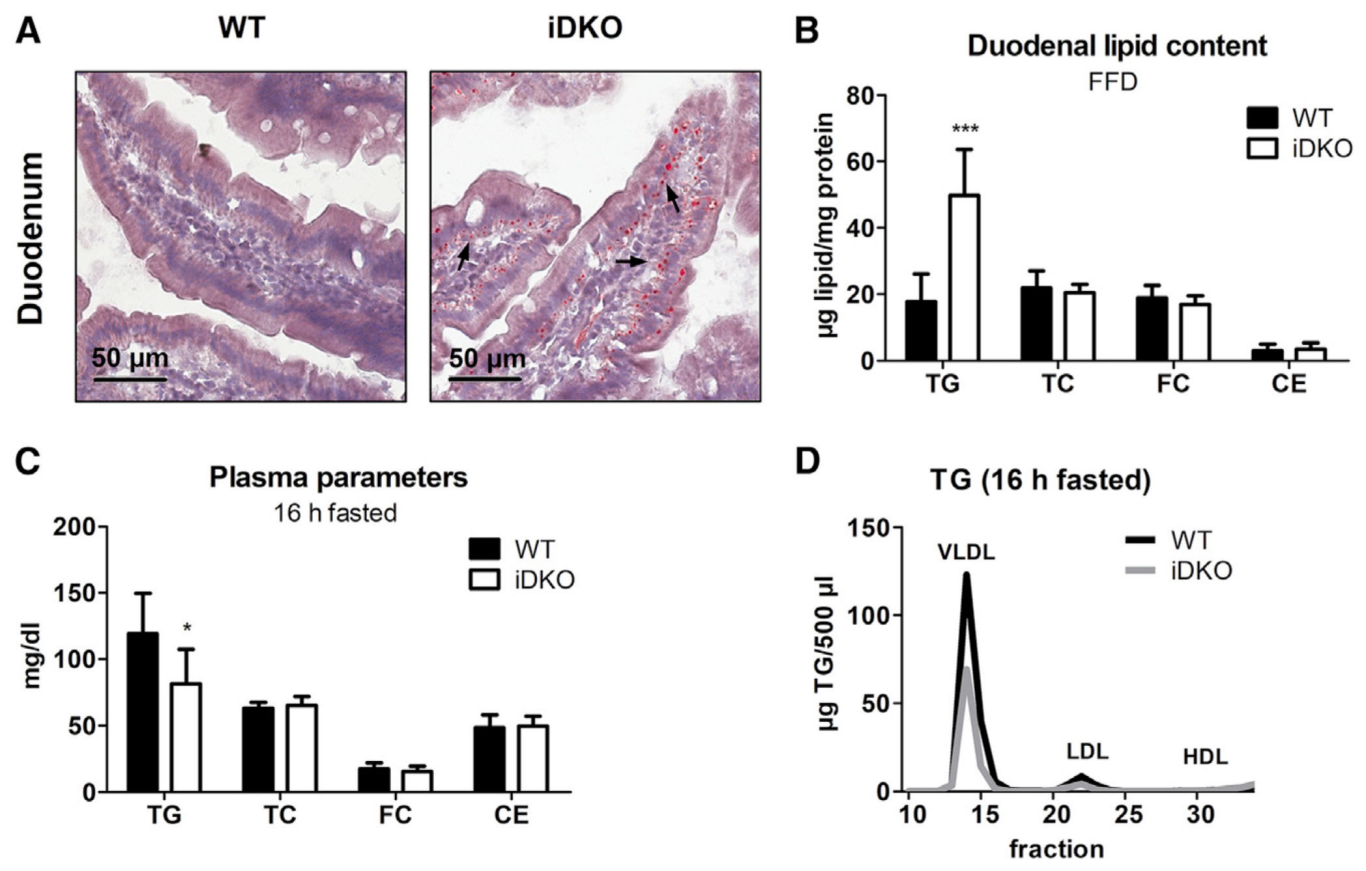
Persistent Increased Lipid Accumulation in the Intestine of iDKO Mice upon Restriction to Endogenous Lipid Sources (A and B) Histological (A) and biochemical lipid (B) analysis in the duodena of WT and iDKO mice (n = 6 or 7) after 5 weeks of FFD feeding (refed state). (C and D) Plasma lipid parameters (C) and lipoprotein profiles (D) in 16 h fasted mice (n = 6–9) fed chow diet. Data represent mean + SD. Magnification, 40×; scale bar, 50 μm. Arrows indicate cLDs on the basolateral pole of the enterocyte. *p < 0.05 and ***p ≤ 0.001. CE, cholesteryl esters; FC, free cholesterol; HDL, high-density lipoprotein; LDL, low-density lipoprotein; TC, total cholesterol; TG, triglycerides; VLDL, very low density lipoprotein. See also Figure S6.

To strengthen our findings, we restricted mice to endogenously produced fats by feeding a fat-free diet (FFD) for 5 weeks. Augmented cLD accumulation (Figure 5A) and 2.8-fold increased TG concentrations (Figure 5B) in the duodenum of FFD-fed iDKO mice were in line with our previous results. However, increased content of starch and sugar in the FFD might also cause increased *de novo* lipogenesis, forming another lipid pool as substrate for ATGL. Although mRNA expression levels of genes involved in *de novo* lipogenesis were comparable (*Acc*) or even reduced (*Fasn*, *Scd-1*, *Srebp1c*) in iDKO duodena, we observed upregulated *Dgat2* and *Mttp* mRNA levels (Figure S6A). Unchanged *Dgat1* but increased *Dgat2* mRNA expression indicate TG synthesis on the cLD surface rather than increased *de novo* lipogenesis because of the different functions of DGAT1 and DGAT2 (Wilfling et al., 2013).

Next, we analyzed intestinal lipid levels after prolonged fasting. Compared with fed conditions (65 μg TG/mg protein; Figure 1C), duodenal TG concentrations in WT mice were markedly reduced after 4 h (32 μg TG/mg protein; Figure 1D) and 16 h of fasting (7 μg TG/mg protein; Figure 1E), indicating that the SI plays a major role in maintaining whole-body lipid homeostasis during starvation. Indeed, whereas body weight remained comparable between the genotypes (Table 1), 16 h fasted iDKO mice displayed a 32% decrease in plasma TG concentrations compared with WT mice (Figure 5C; Table 1), mainly because of reduced VLDL-TG levels (Figure 5D). Unchanged VLDL secretion after tyloxapol injection (Figure S6B) suggests that accelerated lipid uptake from the basolateral side of the enterocyte might be responsible for decreased plasma TG.

### Loss of Intestinal ATGL/CGI-58 Alters Expression of Other Lipases

Finally, as the most striking differences among all genotypes were observed in the very proximal part of the SI, we analyzed the expression profiles of other potential lipases. As the observed increase in lipid accumulation in iDKO mice shifted from the jejunum to the duodenum in the refed state (Figure 1C), we investigated whether downregulated gene expression of other lipases may contribute to augmented duodenal lipid accumulation or if upregulation of lipid hydrolases may attenuate lipid mass in the jejunum.

**Figure 6:**
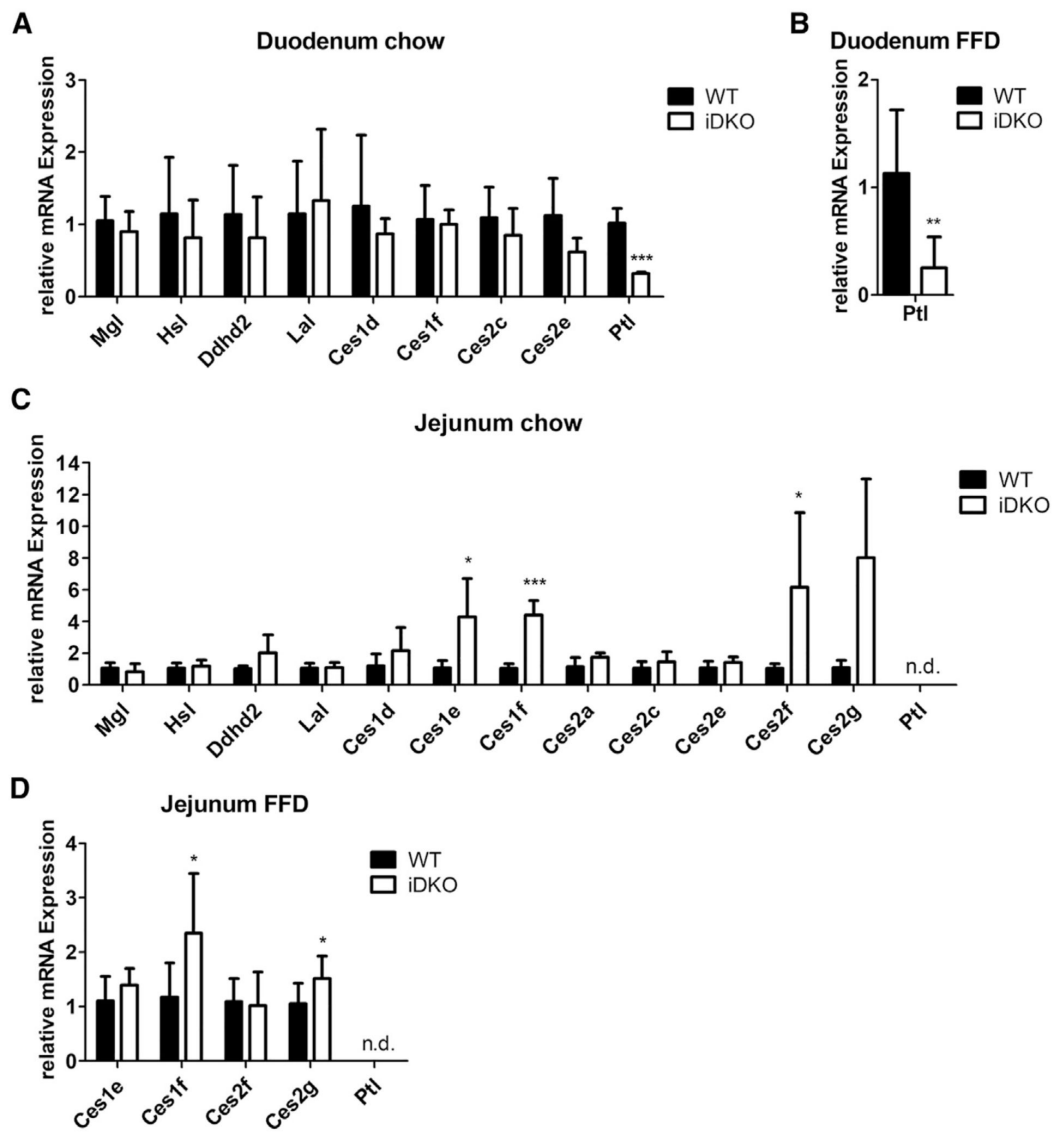
Alterations in mRNA Expression of Potential Lipases in iDKO Mice (A and B) mRNA expression of enzymes involved in neutral (Mgl, Hsl), acidic (Lal), and alkaline (Ptl) lipolysis and other potential TG hydrolases in the duodena of refed WT and iDKO mice fed (A) chow diet or (B) fat-free diet (FFD). (C and D) Jejunal mRNA expression of lipases in the refed state of mice fed chow diet (C) or FFD for 5 weeks (D). Cyclophilin A served as reference gene. Data represent mean + SD (chow, n = 4 or 5; FFD, n = 6 or 7). *p < 0.05, **p ≤ 0.01, and ***p ≤ 0.001. *Ces*, carboxylesterase; *Ddhd2*, DDHD domain-containing 2; *Hsl*, hormone-sensitive lipase; *Lal*, lysosomal acid lipase; *Mgl*, monoglyceride lipase; n.d., not detectable; *Ptl*, pancreatic lipase.

Whereas mRNA expression of the cLD-associated hormone-sensitive lipase (HSL) and monoglyceride lipase (MGL) as well as some endoplasmic reticulum-associated lipases of the carboxylesterase (Ces) family remained comparable between the genotypes, duodenal expression of pancreatic lipase (*Ptl*) was significantly reduced by 70% in chow dietfed (Figure 6A) and FFD-fed iDKO mice (Figure 6B). In accordance with previous data (Mahan et al., 2001), expression of *Ptl* was undetectable in the jejunum of WT and iDKO mice (Figures 6C and 6D). Although expression levels of neutral and acid lipases were comparable in the jejuna of both genotypes, mRNA levels of *Ces1e*, *Ces1f*, and *Ces2g* were significantly increased in chow diet-fed iDKO mice in the refed state (Figure 6C). Although less pronounced, we observed a similar pattern in FFD-fed mice (Figure 6D). A recent study has shown that Ces2c strongly regulates intestinal lipid metabolism and systemic energy homeostasis (Maresch et al., 2018). Whether any other Ces protein is involved in physiological lipid hydrolysis in the SI has to be addressed in future studies.

## Discussion

To prevent FA-driven lipotoxicity, cells esterify absorbed FAs into TGs (Listenberger et al., 2003; Zhu et al., 2009). It has been proposed that (depending on the FA origin) enterocytes store lipids in two different dynamic storage pools: one pool consisting of dietary (apically absorbed) lipids and the second pool containing lipid precursors derived from basolateral uptake. Although alimentary lipids are used primarily for CM synthesis, only a few FAs taken up from the basolateral side are incorporated into CMs (Mansbach and Dowell, 1992) but are suggested to serve other functions such as PL biosynthesis, FA β-oxidation, and signaling (Storch et al., 2008; Trotter and Storch, 1991; Gangl and Ockner, 1975; Gangl and Renner, 1978; Obrowsky et al., 2013). A very recent publication highlighted the importance of basolaterally absorbed lipids on maintaining CM synthesis and secretion of apically absorbed lipids (Li et al., 2019). However, the mechanisms accountable for the mobilization of apically or basolaterally derived lipids remained elusive. We investigated whether ATGL and its coactivator CGI-58, which together mediate the initial step in adipocyte lipolysis (Zimmermann et al., 2004), contribute to the breakdown of one or both of these transient lipid pools. Our results revealed that ATGL and CGI-58 are critically involved in the catabolism of cLDs formed upon re-absorption of lipids from the basolateral side of the enterocyte but not in the hydrolysis of apically derived lipids. This conclusion is drawn from results in iDKO mice including (1) unaffected CM synthesis and circulating lipid concentrations, (2) enterocyte cLD formation 2 h but not 30 min after oral lipid load, (3) increased intestinal accumulation of basolaterally applied lipids, and (4) persistent increased intestinal lipid accumulation despite restriction of dietary fat or after prolonged fasting.

In agreement with previous studies in mice solely lacking ATGL (Obrowsky et al., 2013) or CGI-58 (Xie et al., 2014) in the SI, iDKO accumulated intestinal cLDs to a greater extent than WT mice. Unchanged plasma parameters under various nutritional conditions and comparable CM secretion in iDKO and WT mice are consistent with the finding that hepatic ATGL deletion did not affect VLDL secretion (Wu et al., 2011). In fact, decreased VLDL secretion in whole-body ATGL-KO mice (Haemmerle et al., 2006) is due to reduced FA delivery from the WAT to the liver. CGI-58-mediated TG hydrolysis was suggested to be necessary for efficient lipoprotein secretion in an ATGL-independent manner because lack of intestinal CGI-58 led to reduced plasma TG concentrations (Xie et al., 2014). However, we failed to reproduce these findings in Cgi-58 iKO and iDKO mice. One reason for these contradictory results might be the dietary composition, as Xie et al. (2014) used western type HFD (40% energy from fat; 20.7% [w/w] lard, 0.2% [w/w] cholesterol), whereas we fed mice with HF/HCD (60% energy from fat; 34% [w/w] crude fat, 1% [w/w] cholesterol). These observations, however, indicated that at least one enzyme responsible for cLD hydrolysis to provide FFA for lipoprotein secretion is still elusive. Within enterocytes, cLD catabolism can also be conducted by lysosomal lipolysis. Because LAL affects VLDL synthesis in the liver (Radović et al., 2016), lysosomal acid lipase (LAL) might be a potential candidate for supplying lipid precursors for CM synthesis, rather than ATGL and CGI-58.

CM synthesis is a rapid process, occurring within 12 min in rat jejunum, with cLDs appearing in the endoplasmic reticulum and the Golgi apparatus already 1 min after intra-jejunal injection (Jersild, 1966). Because CM clearance also occurs quite fast in mice with almost complete degradation after 15 min (Quiroga et al., 2012), it is plausible that the SI participates in the clearance of CM remnants from the basolateral side. Interestingly, orally administered lipids excessively accumulated only after 2 h, but not 30 min post-gavage in iDKO mice. This long latency period for augmented cLD accumulation in iDKO enterocytes renders the process of apical uptake of alimentary FAs, their re-esterification in the endoplasmic reticulum, and their storage in the transient cLD pool prior to ATGL/CGI-58-mediated mobilization for CM secretion highly implausible. A re-uptake of CM-secreted dietary lipids from the bloodstream is more likely, causing increased accumulation of cLDs in the SI of iDKO mice. Indeed, the SI is capable of taking up CM remnants basolaterally, as only 71% of esterified oleate stored in the rat intestinal mucosa is of dietary origin (Mansbach and Dowell, 1995). It has been suggested that this process is receptor mediated and most pronounced in the proximal SI (Soued and Mansbach, 1996), which correlates with the increase in radioactively labeled lipids in the duodenum of iDKO mice. Interestingly, 20% of fluorescently labeled (BODIPY-containing) cLDs in the SI of iDKO mice 2 h post-gavage did not colocalize with PLIN3, which is upregulated in the early phase of dietary lipid absorption. This might be attributable to either delayed PLIN3 production, at least slower than the basolateral lipid uptake, or actually reflect PLIN2-positive cLDs, as the proteome of basolaterally derived cLDs might differ from the cytoplasmic storage pool of apically absorbed lipids. Generally, expression of PLIN2 is upregulated upon a chronic lipid challenge such as a high-fat diet (Lee et al., 2009), which argues against the presence of PLIN2 on BODIPY-LDs in our chow diet-fed iDKO mice.

Results from our experiments further highlighted the contribution of hepatocytes on sustaining intestinal lipid homeostasis. As intestinal lipid absorption, CM production, and CM clearance occur quite fast in mice, results from different time points implicate a close interplay between intestinal and hepatic lipid metabolism. Reduced hepatic lipid concentrations 30 min postgavage in iDKO mice might be accountable to slightly delayed gastric emptying, which in turn leads to retarded lipid supply to the liver without affecting intestinal lipid storage. A similar plasma lipid profile in fasted iDKO mice without a dietary trigger ascribes decreased circulating TGs to retarded VLDL synthesis rather than altered intestinal lipid secretion.

Comparable circulating lipid levels in the other experiments indicated an equilibrium of intestinal and hepatic lipoprotein secretion. Interestingly, 1 h after basolateral administration (i.v.) of ^3^H-OA, but not ^3^H-VLDL, hepatic radioactivity was drastically increased in iDKO mice without affecting circulating or intestinal counts. Substrates injected via the retroorbital plexus either first bypass the SI via mesenteric arteries and reach the liver via the portal vein or directly enter the liver via the hepatic artery (Encapsula NanoSciences, 2012). Supposing that the substrate primarily targets the liver via the hepatic artery, hepatocytes usually take up circulating FA rather than VLDL particles (Choi and Ginsberg, 2011). However, WT mice displayed comparable uptake rates of both substrates (6.6% of OA versus 5.7% of VLDL). Assuming that i.v. administered substrates initially get processed by the SI before being shuttled to the liver, it is likely that 1 h was not long enough to process lipoprotein particles (^3^H-VLDL) within the enterocyte. This highlights a role of intestinal ATGL/CGI-58 in processing basolaterally applied FAs, thereby affecting hepatic lipid homeostasis by a yet unknown mechanism. Drastically decreased hepatic radioactivity observed 24 h post-injection of ^3^H-VLDL, but not ^3^H-OA, indicates a competition of lipoprotein uptake between the SI and the liver, as suggested previously (Mansbach and Dowell, 1995). However, it remains enigmatic in which form the tracer is delivered to the tissues 24 h after i.v. administration of ^3^H-VLDL. We showed that ^3^H-OA does not get processed and incorporated into lipoproteins 24 h post-injection after a 12 h fasting period but rather circulates in form of FFA in the plasma. However, it is worth mentioning that active lipoprotein lipase (LPL) in the plasma might have already degraded plasma TGs until the time of analysis, thereby falsifying the distribution of the radioactive tracer. Clearance of 95% of the injected tracer within 1 h and shuttling of <10% to the liver and intestine suggest a dynamic distribution among organs besides the enterohepatic system. Although our study highlights the close connection between gut and liver lipid metabolism, further studies are required to completely understand the interplay between SI, lipoprotein metabolism, and hepatocytes. Detailed flux analyses tracing lipids and their distribution among metabolically active organs are needed to elucidate their role in whole-body lipid homeostasis.

The sequential meal study in humans demonstrated that lipids ingested with an initial meal appear in the plasma shortly after ingestion of a second meal, supporting the existence of a transient lipid storage pool (Fielding et al., 1996; Jackson et al., 2002). If ATGL and CGI-58 were involved in the hydrolysis of this lipid pool, secretion of primary ingested lipids would be diminished in iDKO mice after ingestion of the subsequent meal. Plasma concentrations of primary ingested lipids upon administration of a secondary oil bolus, however, were unaltered among the genotypes, again emphasizing that intestinal ATGL and CGI-58 do not affect secretion of dietary lipids into the periphery. The absence of the sequential meal effect in WT mice raises the question of the relevance of this phenomenon in mice. Lipids from the first but not the second meal accumulated to a higher extent in the SI of iDKO mice and the distribution of the second tracer resembled an early absorptive phase in iDKO mice with reduced gastric emptying and delayed appearance of the lipid in the periphery. This result might be due to (1) an overload of lipids, leading to saturation of the intestinal storage capacity; (2) lipids of the second meal bypassing cytosolic storage and/or hydrolysis and being directly secreted into the circulation independent of ATGL/CGI-58; or (3) potentiated lipid-triggered delay in gut transit (data not shown) with insufficient time for lipid re-absorption at the basolateral side and accumulation as cLDs as putative substrates for intestinal ATGL and CGI-58.

Uptake and augmented intracellular TG accumulation were independent of nutritional challenges, but solely mediated by the loss of intestinal ATGL and CGI-58. Given that both proteins selectively hydrolyze basolaterally absorbed lipids, which predominantly enter the FA β-oxidation pathway (Storch et al., 2008; Ho et al., 2002; Gangl and Ockner, 1975), the generated FFAs are most likely used for energy production. In accordance with previous studies on hepatic CGI-58 (Brown et al., 2010) as well as hepatic (Ong et al., 2011) and intestinal (Obrowsky et al., 2013) ATGL, we observed an impaired FA oxidation in iDKO enterocytes. Although analysis of intestinal FA oxidation *in vivo* might help identify the source of FA taken up by the basolateral side of the enterocyte, entire lipoprotein particles are unlikely taken up by receptor-mediated endocytosis. It is rather likely that TG-rich lipoproteins are cleaved by LPL to release FAs, which are then taken up by enterocytes, re-esterified into TGs, hydrolyzed by ATGL/CGI-58, and used for FA oxidation. The importance of LPL cleavage was highlighted in previous publications showing that only CM remnants, but not CMs, can be absorbed basolaterally (Soued and Mansbach, 1996). It is therefore difficult to distinguish the origin of FFA used for FA oxidation in isolated enterocytes, however, exposure of isolated cells with VLDL particles would not reflect physiological conditions.

Basolaterally absorbed lipids can also be shuttled to PL synthesis in the enterocyte (Storch et al., 2008; Ho et al., 2002; Gangl and Ockner, 1975). Incorporation of radioactive tracers into the PL fraction, however, was unaffected in iDKO mice, most likely because of the stereoselectivity of ATGL. The lipolytic products generated by ATGL (sn-1,3 DG and, upon stimulation by CGI-58, sn-2,3 DG) are rather hydrolyzed by HSL (sn-1,3 DG) or re-esterified by DGAT enzymes (sn-2,3 DG) but cannot directly enter the PL synthesis pathway (Eichmann et al., 2012). Thus, upon re-esterification, storage in a basolaterally derived lipid pool, and ATGL hydrolysis, FFAs are rather used for β-oxidation or PPARα signaling.

In contrast to the fasted state, refed iDKO mice displayed increased TG accumulation only in the duodenum, suggesting an upregulation of other lipase(s) in the jejunum. Whether the drastic increase in mRNA expression of several Ces proteins in the jejunum of iDKO mice contributes to this effect remains enigmatic. Although Ces1f preferentially hydrolyzes short-chain FA-containing TGs in adipocytes, the *in vivo* role of this enzyme in lipolysis or intestinal lipid metabolism is unknown (Okazaki et al., 2006). In contrast to adipose tissue, intestinal mRNA expression of *Ces1f* increases upon refeeding (Okazaki et al., 2006). Therefore, upregulation of *Ces1f* or *Ces2g*, which is markedly increased in mouse steatotic livers (Jones et al., 2013), might take over the role of ATGL, thereby preventing augmented jejunal TG levels. In the duodenum, mRNA expression of potential candidates (including Hsl and Ces family members) were unaltered. Whether the drastically reduced mRNA expression of *Ptl* contributes to the increase in duodenal TG content of iDKO mice is currently unknown. The impact of PTL, Ces1e, Ces1f, Ces2g, or other Ces family members on intestinal lipid metabolism in iDKO mice needs to be evaluated in future studies.

In summary, this study demonstrates that intestinal ATGL and CGI-58 are not involved in the catabolism of alimentary lipids absorbed at the apical side. In contrast, these two proteins contribute to the hydrolysis of re-absorbed TGs originating from basolateral absorption in enterocytes, which are not destined for CM synthesis. Future studies elucidating this process, the close interplay between the SI and hepatocytes, and identifying the yet unknown enzyme responsible for degradation of the apically derived lipid pool are needed to fully understand intestinal lipid metabolism.

## Star★Methods

### Key Resources Table

**Table:** 

REAGENT or RESOURCE	SOURCE	IDENTIFIER
Antibodies		
ATGL Antibody	Cell Signaling Technology	Cat#2138; RRID:AB_2167955
ABHD5 monoclonal antibody (M01), clone 1F3	Abnova	Cat#H00051099-M01; RRID:AB_509070
Anti-Perilipin-3, C terminus Antibody	Merck KGaA	Cat#ABS482;
Polyclonal Rabbit anti-Mouse Immunoglobulins (HRP)	Dako	Cat#P0260; RRID:AB_2687969
Goat anti-Rabbit IgG (HRP)	Thermo Fisher Scientific	Cat#31460; RRID:AB_228341 LOT#QG221919
Goat anti-Rabbit IgG, Alexa Fluor® 594	Thermo Fisher Scientific	Cat#A11037; RRID:AB_2534095 LOT#1310680
Critical Commercial Assays		
Triglycerides FS	DiaSys Diagnostic Systems GmbH	Cat#157609910023
Cholesterol FS	DiaSys Diagnostic Systems GmbH	Cat#113009910023
Free Cholesterol FS	DiaSys Diagnostic Systems GmbH	Cat#113609910930
Free Glycerol Reagent	Merck KGaA	Cat#F6428
NEFA-HR(2)	Wako Chemicals GmbH	Cat#434-91795, Cat#436-91995, Cat#270-77000
Bio Rad Protein Assay	Bio-Rad Laboratories	Cat#500-0112
Experimental Models: Organisms/Strains		
Mouse: WT Atgl^flox/flox^: B6.129-Pnpla2^tm1Eek^	Erin Kershaw	Cat#JAX: 024278; RRID: IMSR_JAX: 024278
Mouse: Atgl iKO Atgl^flox/flox^/Villin-Cre^(ER-T2)^	Obrowsky et al., 2013	N/A
Mouse: WT Cgi-58^flox/flox^: B6.129-Abhd5^tm1.1Rze-flox^	Guenter Haemmerle	N/A
Mouse: Cgi-58 iKO Cgi-58^flox/flox^/Villin-Cre^(ER-T2)^	This paper	N/A
Mouse: WT Atgl^flox/flox^/Cgi-58^flox/flox^	This paper	N/A
Mouse: Atgl/Cgi-58 iDKO Atgl^flox/flox^/Cgi-58^flox/flox^/Villin-Cre^(ER-T2)^	This paper	N/A
Oligonucleotides		
See Table S1 for list of all primer sequences used for qRT-PCR		N/A
Software and Algorithms		
Graph Pad Prism 5	GraphPad Software	https://www.graphpad.com
ImageJ	ImageJ	https://imagej.net
VisiView acquisition software	Universal Imaging, Visitron Systems	N/A

### Lead Contact and Materials Availability

Further information and requests for resources and reagents should be directed to and will be fulfilled by the Lead Contact, Dagmar Kratky (dagmar.kratky@medunigraz.at). All materials, including iDKO mice, transferred will require Material Transfer Agreements (MTAs) between the Medical University of Graz and the respective institution.

### Experimental Model and Subject Details

Mice carrying a LoxP-modified *Atgl* or *Cgi-58* allele (backcrossed onto the C57BL/6J background) were generated in the laboratories of Erin Kershaw (Obrowsky et al., 2013) and Guenter Haemmerle (Zierler et al., 2013), respectively. To produce intestine-specific Atgl (Atgl iKO) (Obrowsky et al., 2013) and Cgi-58 KO (Cgi-58 iKO) mice, floxed mice were interbred with transgenic mice expressing Cre recombinase under the control of the intestinal epithelial cell-specific villin promoter (Madison et al., 2002). Intestine-specific Atgl/Cgi-58 double KO (iDKO) mice were generated by crossing Atgl iKO and Cgi-58 iKO mice. All experiments were performed using female mice aged between 13 and 14 weeks, unless stated otherwise. Age- and sex-matched Atgl/Cgi-58 iDKO and Atgl^flox/flox^/Cgi-58^flox/flox^ WT littermates were maintained in a temperature-controlled environment with unlimited access to food and water in a regular light-dark cycle (12 h/12 h). Mice were fed a standard chow diet (11.9% caloric intake from fat; Altromin, Lage, Germany) or challenged with either high fat/high cholesterol [HF/HCD; 34% (w/w) crude fat, 1% (w/w) cholesterol; Ssniff®, Soest, Germany] or fat-free diet [FFD; 45% (w/w) starch, 16.8% (w/w) sugar, 0.2% (w/w) crude fat; Ssniff®, Soest, Germany]. Mice were sacrificed after an indicated fasting period (4 h or 16 h) or in the refed state (12 h fasting, 2 h refeeding). Mice fed HF/HCD were housed individually and food intake as well as fecal output were monitored over a period of 3 days. Food intake was calculated as g/day/mouse. To determine fecal fat weight, feces of HF/HCD-fed mice were collected, weighed, and lipids were extracted according to Folch. Lipid extracts were weighed and the ratio of lipid weight to feces weight was calculated.

All experiments were performed in accordance with the European Directive 2010/63/EU and approved by the Austrian Federal Ministry of Education, Science and Research (Vienna, Austria; BMWFW-66.010/0057-WF/V/3b/2015).

### Method Details

#### Plasma and Tissue Lipid Analyses

Blood was collected from the retrobulbar plexus and centrifuged for 7 min at 5,200 *x g* and 4°C for plasma isolation. Plasma triglyceride (TG), total cholesterol (TC), free cholesterol (FC), free glycerol (FG), and non-esterified FA (NEFA) concentrations were assayed using enzymatic kits according to manufacturer’s protocols (DiaSys, Holzheim, Germany; Merck KGaA, Darmstadt, Germany; Wako Chemicals GmbH, Neuss, Germany). Plasma CE concentrations were calculated by subtracting FC from TC. To separate lipoproteins, a pool of 200 μl plasma per genotype was subjected to fast protein liquid chromatography (Pharmacia P-500) equipped with a Superose 6 column (Amersham BioscienCes, Piscataway, NJ).

For intestinal lipid analysis, the SI was divided into three equal parts. Considering that the duodenum accounts for approximately 11% of the length, duodenal samples were taken from the very proximal part of the small intestine. For experiments in the jejunum, we took the middle part, representing the proximal jejunum. Ileal samples were taken from the very distal part of the small intestine to ensure the expression of bile acid transporters. Lipids were isolated from mucosal scrapings of the three parts of the SI and from livers by Folch extraction. Briefly, a chloroform/methanol (2:1) solution in 20-fold excess was added to the tissue lysates, which were then rotated for 2 h at room temperature. After centrifugation at 3,200 *x g* for 15 min, 0.2 volume parts of PBS were added to the supernatant. Samples were vortexed and centrifuged for 15 min at 800 *x g*. The lower phase was taken and dried under a stream of nitrogen. One hundred μl of 2% Triton X-100 in chloroform were added and dried under nitrogen gas. Thereafter, the samples were dissolved in 100 μl ddH_2_O, and TG, TC, and FC concentrations were measured using the above mentioned kits. CE concentrations were calculated by subtracting FC from TC. All values were normalized to protein concentrations.

#### CM Secretion, CM Size, and VLDL Secretion

For CM secretion, chow diet-fed mice were fasted for 16 h and injected with 500 mg tyloxapol/kg body weight (Merck KGaA; Darmstadt, Germany) to inhibit peripheral lipolysis. Thirty minutes later, mice were gavaged with 200 μl olive oil as a substrate to trigger CM synthesis (Jackson et al., 2002). Blood was taken prior to the injection as well as 1, 2, 3, and 4 h post olive oil gavage. Plasma TG and TC concentrations were measured as described above. For analysis of CM size, mice were fasted for 4 h prior to injection of tyloxapol (500 mg/kg body weight). One hour later, mice were administered an olive oil bolus (200 μl) and blood was collected 90 min post gavage. Plasma was obtained via centrifugation (7 min at 5,200 *x g* and 4°C) and pooled for each genotype. Samples (125 μl) were mixed with 280 μl of buffer (PBS, 2 mM benzamidine, 4 M KBr) and 4 mL of 0.9% NaCl before ultracentrifugation for 45 min. CMs were isolated from the upper phase and size was measured in technical triplicate by light scattering (Malvern Zetasizer, Malvern Panalytical GmbH, Kassel, Germany).

VLDL secretion was determined in 16 h-fasted mice after tyloxapol injection (500 mg/kg body weight). Blood was taken prior to injection as well as 1, 2, 4, and 6 h post-injection.

#### Postprandial TG Secretion

Postprandial TG secretion was performed as described previously (Xie et al., 2014). Briefly, Cgi-58 iKO and control mice on HF/HCD for six weeks were fasted overnight (16 h) and injected with 500 mg tyloxapol/kg body weight. Thirty minutes later, mice were gavaged with 500 μl olive oil and blood was taken prior to the gavage as well as 2, 4 and 6 h post-gavage.

#### Histology and ORO Staining

Small intestines and livers were fixed in 4% neutral-buffered formalin for 24 h and stored in 30% sucrose before cryosectioning. Intestinal and hepatic sections (5 μm) were cut (HM 560 Cryo-Star; Microm International GmbH, Walldorf, Germany) and stained with ORO and Mayer’s hematoxylin to visualize neutral lipids and nuclei, respectively. Images were taken in 40x magnification using the Aperio ScanScope AT microscope (Leica Biosystems Nussloch GmbH, Nussloch, Germany).

#### BODIPY® Gavage and Immunofluorescence Staining

Mice were fasted for 16 h prior to an oral administration of 100 μl corn oil containing BODIPY®-C_16_ (BODIPY® FL C_16_; Molecular Probes Europe BV, Leiden, Netherlands; 1 μg/g body weight). In contrast to all other experiments, we decreased the volume to 100 μl to reduce toxic side effects from DMSO and to avoid dilution of the fluorescent signal. Thirty min and 2 h post-gavage, mice were euthanized, intestines were fixed in 4% neutral-buffered formalin for 4 h, and stored in 30% sucrose. Cryosections (5 μm) were mounted using Vectashield® Mounting Medium with DAPI (Vector Laboratories, Inc., Burlingame, CA) or used for immunofluorescence staining with PLIN3 antibody.

For co-staining with perilipin 3 (PLIN3), cryosections were rehydrated in Tris-buffered saline (TBS) and blocked with 0.05% TBST (TBS plus 0.05% Tween 20) containing 10% anti-goat serum. Sections were incubated overnight at 4°C with the primary antibody against PLIN3 (abs482; 1:200; Merck KGaA, Darmstadt, Germany), followed by incubation with the corresponding Alexa Fluor® 594-labeled secondary antibody for PLIN3 (A11037; 1:250; Thermo Fisher Scientific, Waltham, MA). Sections were mounted using Vectashield® Mounting Medium with DAPI and images were captured with a confocal spinning disk microscope (Zeiss Axio Observer.Z1, Goettingen, Germany) equipped with a 100x objective lens (Plan-Fluor x100/1.45 Oil, Zeiss), a motorized filter wheel (CSUX1FW, Yokogawa Electric Corporation, Tokyo, Japan) on the emission side, an AOTF-based laser merge module for laser lines 405, 445, 473, 488, 561, and 561 nm (Visitron Systems), and a Nipkow-based confocal scanning unit (CSU-X1, Yokogawa Electric corporation). The BODIPY®- and Alexa Fluor® 594-labeled structures were excited with 488 and 561 laser lines, respectively, and emission was acquired by a CCD camera (CoolSNAP-HQ, Photometrics, Tucson, AZ, USA). VisiView acquisition software (Universal Imaging, Visitron Systems) was used to acquire the imaging data.

#### Western Blotting

Mucosal scrapings were lysed (Precellys; Bertin Instruments, Bretonneux, France) and subsequently sonicated (Labsonic B. Braun, Melsungen, Germany) for 10 s. After centrifugation for 3 min at 18,000 *x g*, protein concentration in the supernatant was estimated according to the method of Lowry (BioRad Laboratories, Hercules, CA). Eighty micrograms of protein of tissue lysates were separated by SDS-PAGE and transferred onto a nitrocellulose or PVDF membrane to detect ATGL (#2138; 1:200; Cell Signaling Technology; Danvers, MA) and CGI-58 (#H00051099-M01; 1:500; Abnova; Taipei City, Taiwan), respectively. Monoclonal anti-mouse β-actin (Santa Cruz Biotechnology, Heidelberg, Germany) was used as loading control. Secondary anti-rabbit (1:2,500) or anti-mouse (1:500) antibodies, conjugated with HRP (Thermo Fisher Scientific, Waltham, MA; Dako, Glostrup, Denmark), were visualized using the ClarityTM Western ECL Substrate Kit (Bio Rad Laboratories; Hercules, CA) on a ChemiDoc MP imaging system (Bio Rad Laboratories; Hercules, CA).

#### RNA Isolation and Quantitative Real-Time PCR

RNA was extracted using peqGOLD TriFast according to the manufacturer’s protocol (Peqlab, Erlangen, Germany) and 2 μg of RNA were reverse transcribed using the High Capacity cDNA Reverse Transcription Kit (Applied Biosystems, Carlsbad, CA). Quantitative real-time PCR was performed on a Roche LightCycler 480 (Roche Diagnostics, Palo Alto, CA) using the GoTaq® qPCR Mastermix (Promega, Madison, WI). Samples (40 ng) were analyzed in duplicate and normalized to the expression of cyclophilin A as house-keeping gene. Expression profiles and associated statistical parameters were determined using the 2^-ΔΔCT^ method.

#### Triglyceride Hydrolase Activity Assay

Tissues were lysed in lysis buffer (100 mM potassium phosphate, 250 mM sucrose, 1 mM EDTA, 1 mM DTT, pH 7), sonicated on ice (3x10 s with 1 min interval), and centrifuged at 1,000 *x g* and 4°C for 10 min. The supernatant was again centrifuged at 20,000 *x g* for 30 min at 4°C and the protein concentration was estimated in the lipid-free infranatant (BioRad Laboratories, Hercules, CA). Fifty micrograms of protein were diluted to a final volume of 100 μl with lysis buffer and used to determine neutral TG hydrolase activity. To measure neutral TG hydrolase activity, the samples were mixed with 100 μL of TG substrate [per sample: 0.3 mM triolein, 0.5 μCi [9,10-^3^H(N)]-triolein (Perkin Elmer, Waltham, MA), 3.5 μg mixed micelles of phosphatidylcholine and phosphatidylinositol (3:1, w:w)]. Each substrate contained NEFA-free BSA at a final concentration of 2% in 100 mM phosphate buffer. After incubation in a water bath for 1 h at 37°C, the reaction was stopped by the addition of 3.25 mL stop solution (methanol:chloroform:n-heptane, 10:9:7, v:v:v) and 1 mL of 0.1 M potassium carbonate (pH 10.5, adjusted with boric acid). The tubes were vortexed for 10-15 s and centrifuged at 800 *x g* for 15 min at 4°C. The radioactivity in 1 mL of the upper phase was determined by liquid scintillation counting and the release of FAs was calculated.

#### Sequential Meal Study

After a 12 h fasting period, mice were gavaged with 200 μl olive oil containing 0.5 μCi [1-^14^C]-triolein (Perkin Elmer, Waltham, MA). Blood was taken 2, 4, and 6 h post-gavage. Six hours after the first bolus, mice were gavaged with 200 μl olive oil containing 2 μCi [9,10-^3^H(N)]-triolein (Perkin Elmer) and blood was taken 2 and 4 h later. Four hours after the second bolus, mice were sacrificed and tissues were collected, lyophilized, and digested in 1 mL of 1 M NaOH. Radioactivity was measured by liquid scintillation counting.

For determination of the lipid distribution into different lipid classes, lipids were extracted from 20 mg of lyophilized tissue using chloroform:methanol (2:1). Lipid extracts were separated using n-hexane:diethylether:acetic acid (70:30:1, v:v:v), corresponding bands for PL, FC, FFA, TG, and CE were cut, and radioactivity was determined by liquid scintillation counting.

#### Apical Lipid Absorption

To study the early absorption phase, overnight-fasted mice (16 h) were gavaged with 200 μl corn oil containing 2 μCi [9,10-^3^H(N)]-triolein (Perkin Elmer, Waltham, MA) and sacrificed 30 min post-gavage. Intestine, stomach, and liver were collected, lyophilized, and radioactivity was determined by liquid scintillation counting.

#### Basolateral Lipid Absorption

To discriminate between FA- or lipoprotein-derived lipids, 5 μCi [9,10-^3^H(N)]-oleic acid (Hartmann Analytics, Braunschweig, Germany) were complexed with Na-oleate and BSA and injected in 200 μl PBS intravenously (i.v.) into WT and iDKO mice (Vujic et al., 2017). For lipoprotein injection, human VLDL (2.44 mg TG) was labeled with 1 μCi [9,10-^3^H(N)]-triolein (Perkin Elmer, Waltham, MA) and administered i.v. (200 μl). Animals were injected at 8 a.m. and sacrificed 1 h later to collect plasma and organs. To trigger VLDL secretion from the liver, mice were injected i.v. at 8 a.m., followed by overnight fasting the same day (8 p.m. – 8 a.m.) and sacrificed at 8 a.m. the following day. Radioactivity in plasma and tissues was determined by liquid scintillation counting.

#### Fatty Acid β-oxidation in Isolated Enterocytes

Isolation of primary enterocytes was performed as described elsewhere (Khalifeh-Soltani et al., 2016). Briefly, the jejunal segment was washed with Buffer A (115 mM NaCl, 5.4 mM KCl, 0.96 mM NaH_2_PO_4_, 26.19 mM NaHCO_3_, 5.5 mM glucose). One end of the intestine was tied and the lumen was filled with Buffer B (67.5 mM NaCl, 1.5 mM KCl, 0.96 mM NaH_2_PO_4_, 26.19 mM NaHCO_3_, 27 mM sodium citrate, 5.5 mM glucose). After incubation for 15 min in 0.9% NaCl at 37°C, the luminal content was discarded and the jejunum was filled with Buffer C (Buffer A plus 1.5 mM EDTA and 0.5 mM DTT). After incubation for 10 min in 0.9% NaCl at 37°C, the luminal content was collected, filtered, and centrifuged at 1,500 *x g* for 5 min at room temperature. All buffers were adjusted to pH 7.4 and aerated wit 95% O_2_ and 5% CO_2_ before use.

Enterocyte pellets were resuspended in 1 mL DMEM containing 0.5 mM carnitine, 100 μM palmitic acid, and 0.4 mCi [1-^14^C]-palmitic acid. The cell culture flask containing a center well with a saturated (50 μl 1 M NaOH) filter was sealed with a rubber stopper and incubated for 90 min at 37° C. The reaction was terminated by the addition of 100 μl 70% perchloric acid and ^14^CO_2_ was trapped for 2 h at 37°C. Radioactivity on the filter paper was counted by liquid scintillation counting. Results were normalized to protein content.

#### Image Processing

Only for illustrative purposes, ImageJ was used for contrast adjustments and to create overlay images of immunofluorescence pictures.

### Quantification and Statistical Analysis

Statistical analyses were performed using GraphPad Prism 5.0 software. Significance was calculated by unpaired Student’s t test or ANOVA followed by Bonferroni post-tests. For statistical analysis of mRNA expression, values were calculated using the 2^-ΔΔCT^ method. Data are presented as mean ± SD for biological replicates or as mean ± SEM for technical replicates (e.g., quantification of cLDs) with the following levels of statistical significance:

*, p < 0.05; **, p ≤ 0.01; ***, p ≤ 0.001. Statistical details for the respective experiments are stated in the figure legends.

### Data and Code Availability

The published article includes all datasets generated or analyzed during this study.

## Supplementary Material

Supplemental Information can be found online at https://doi.org/10.1016/j.celrep.2019.07.030.

Supplement

## Figures and Tables

**Table 1:** Body Weight and Plasma Parameters of Sex- and Age-Matched WT and iDKO Mice

	Chow (16 h Fasted)		Chow (4 h Fasted)		Chow (Refed)	
		
	WT	iDKO	WT	iDKO	WT	iDKO
BW (g)	19.4 ± 1.74	19.2 ± 0.89	21.9 ± 1.95	20.8 ± 2.26	21.2 ± 0.21	20.1 ± 2.15
TG (mg/dL)	119 ± 30.4	81.5 ± 26.0*	85.2 ± 20.4	83.2 ± 19.1	57.2 ± 6.73	53.6 ± 17.6
TC (mg/dL)	63.1 ± 4.09	65.1 ± 6.59	68.1 ± 9.14	58.4 ± 7.81	54.4 ± 7.71	56.0 ± 4.36
FC (mg/dL)	17.3 ± 4.64	15.5 ± 3.89	13.8 ± 3.48	13.5 ± 2.84	18.7 ± 2.25	19.7 ± 2.09
CE (mg/dL)	48.5 ± 9.51	49.6 ± 7.56	53.9 ± 6.60	46.1 ± 6.54*	35.7 ± 5.74	36.3 ± 4.21
FFA (mmol/L)	0.99 ± 0.31	1.02 ± 0.42	0.48 ± 0.36	0.45 ± 0.37	0.20 ± 0.05	0.17 ± 0.02
n	7	9	9	9	5	5
	5 week HF/HCD (4 h fasted)		5 week FFD (refed)		chow 16 h fasted (30 min post-gavage)	
	WT	iDKO	WT	iDKO	WT	iDKO
BW (g)	22.8 ± 2.35	23.7 ± 1.98	22.1 ± 1.07	22.5 ± 0.93	n.d.	n.d.
TG (mg/dL)	54.5 ± 7.53	51.9 ± 6.53	63.7 ± 11.4	57.0 ± 7.72	82.7 ± 10.4	43.2 ± 12.7**
TC (mg/dL)	106 ± 11.4	96.8 ± 9.13	41.0 ± 8.42	43.1 ± 6.66	63.5 ± 7.41	65.9 ± 6.32
FC (mg/dL)	26.7 ± 3.70	22.8 ± 2.78	16.3 ± 2.98	16.3 ± 3.67	11.0 ± 0.72	11.4 ± 0.82
CE (mg/dL)	79.7 ± 7.81	74.0 ± 6.41	24.7 ± 6.49	26.8 ± 3.35	52.5 ± 6.75	54.5 ± 5.51
FFA (mmol/L)	0.58 ± 0.05	0.63 ± 0.10	1.17 ± 0.52	1.13 ± 0.57	0.45 ± 0.10	0.43 ± 0.10
n	5	5	7	7	4	4

Data represent mean ± SD (n = 4–9). BW, body weight; CE, cholesteryl esters; FC, free cholesterol; FFA, free fatty acids; n.d., not determined; TC, total cholesterol; TG, triglycerides.

*p < 0.05 and ** p ≤ 0.01.

## Highlights

- Loss of enterocyte ATGL/CGI-58 causes massive intestinal lipid accumulation
- Dietary lipids for chylomicron synthesis bypass ATGL/CGI-58-mediated catabolism
- Intestinal ATGL and CGI-58 hydrolyze lipids derived from the basolateral side (blood)
- A lipid secretion/re-uptake cycle is present in the small intestine
