# Supplementary material for: ATGL/CGI-58-Dependent Hydrolysis of a Lipid Storage Pool in Murine Enterocytes
Source: Cell Rep. Author manuscript; Available in PMC 2019 Aug 28. (PMC6713565; doi:10.1016/j.celrep.2019.07.030)
Supplement: Supplement [file EMS84137-supplement-Supplement.pdf]

Cell Reports, Volume *28*

Supplemental Information

ATGL/CGI-58-Dependent Hydrolysis of a Lipid Storage Pool in Murine Enterocytes

Melanie Korbelius, Nemanja Vujic, Vinay Sachdev, Sascha Obrowsky, Silvia Rainer, Benjamin Gottschalk, Wolfgang F. Graier, and Dagmar Kratky

Supplemental Figures

Figure S1

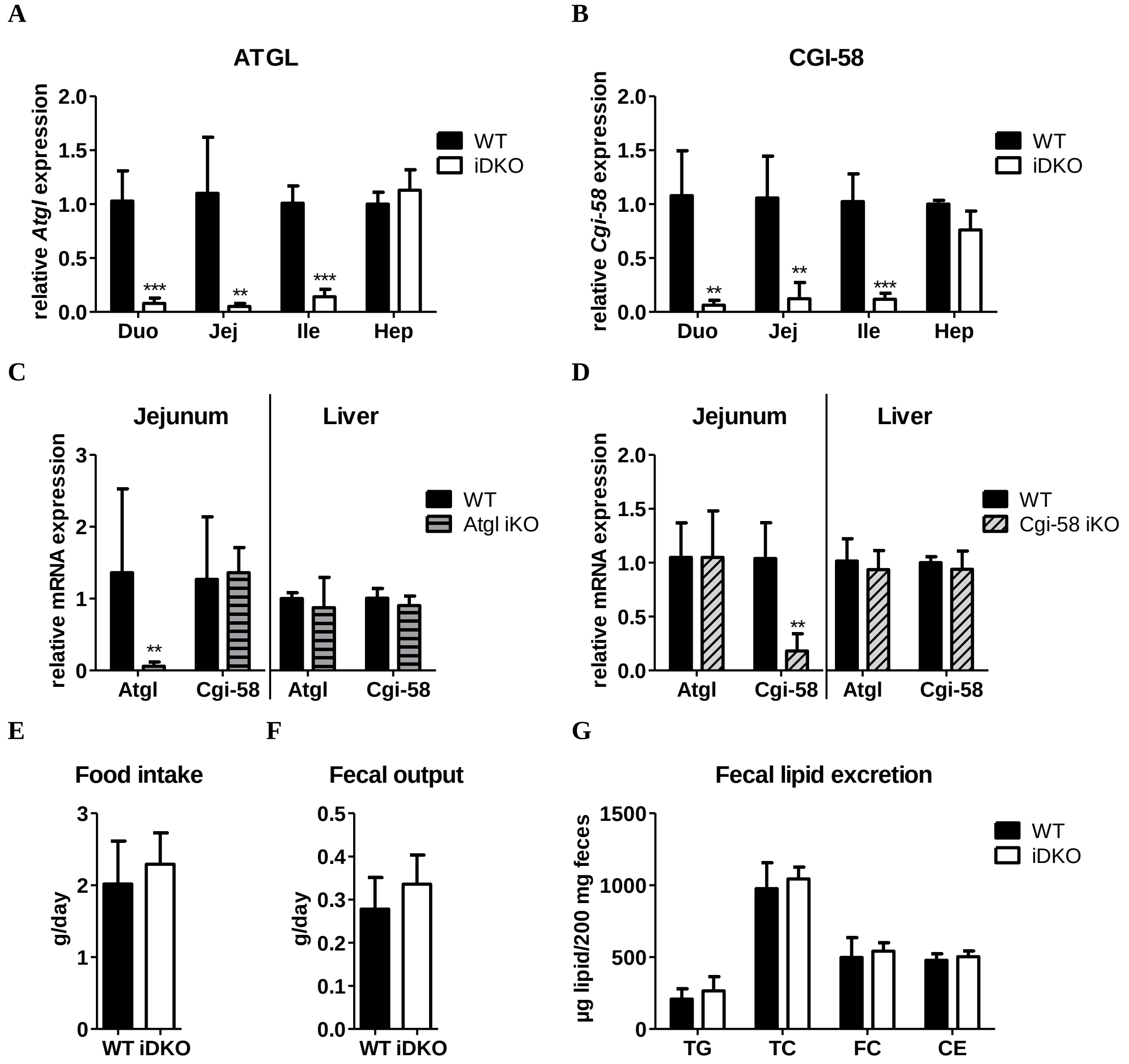

Figure S1: Efficient knockout of intestinal ATGL and CGI-58, related to Figure 1. Intestinal and hepatic gene expression of A) *Atgl* and B) *Cgi-58* in iDKO mice (n=4). *Atgl* and *Cgi-58* mRNA expression in jejunum and liver of C) Atgl iKO (n=3-4) and D) Cgi-58 iKO mice (n=4). E) Food intake was monitored for three consecutive days in iDKO and WT mice fed HF/HCD for 5 weeks (n=5). Feces were collected daily and lipids were extracted from 200 mg pulverized feces using the Folch method. F) Fecal output and G) fecal lipid excretion in HF/HCD-fed WT and iDKO mice (n=5). Data represent mean + SD. ** $p \leq 0.01$ *** $p \leq 0.001$. Duo, duodenum; Jej, jejunum; Ile, ileum; Hep, hepar (liver); TG, triglycerides; TC, total cholesterol; FC, free cholesterol; CE, cholesteryl esters.

Figure S2

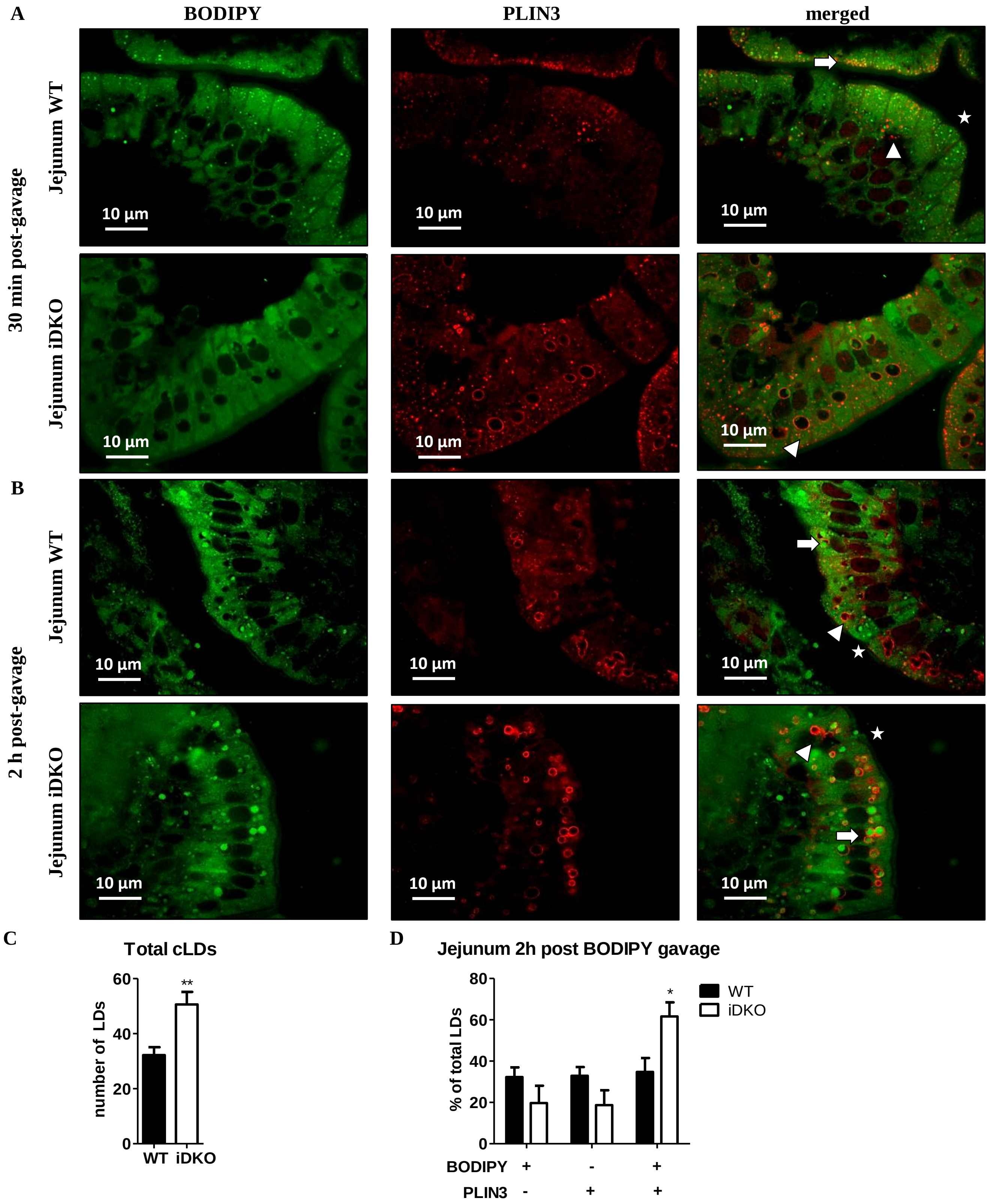

Figure S2: Different fates of lipids 30 min and 2 h post-gavage in iDKO mice, related to Figure 2. Perilipin 3 (PLIN3; red) immunofluorescence staining in the jejunum of BODIPY®-gavaged (green) WT and iDKO mice A) 30 min and B) 2 h post-gavage. C) Total number of lipid droplets (LDs) in the jejunum of WT and iDKO mice 2 h post BODIPY® gavage. D) Relative amount of cLDs containing BODIPY®, coated with PLIN3, and BODIPY®-containing PLIN3-coated vesicles 2 h post gavage. LDs were quantitated by counting of multiple fluorescence micrographs (n=5). Data represent mean + SEM. * $p < 0.05$; ** $p \leq 0.01$. Arrows indicate cLDs originating from BODIPY® -labeled FA, which colocalize with PLIN3; arrowheads indicate endogenous cLDs coated with PLIN3; stars indicate BODIPY®-containing cLDs, which do not colocalize with PLIN3. Magnification, 100x.

Figure S3

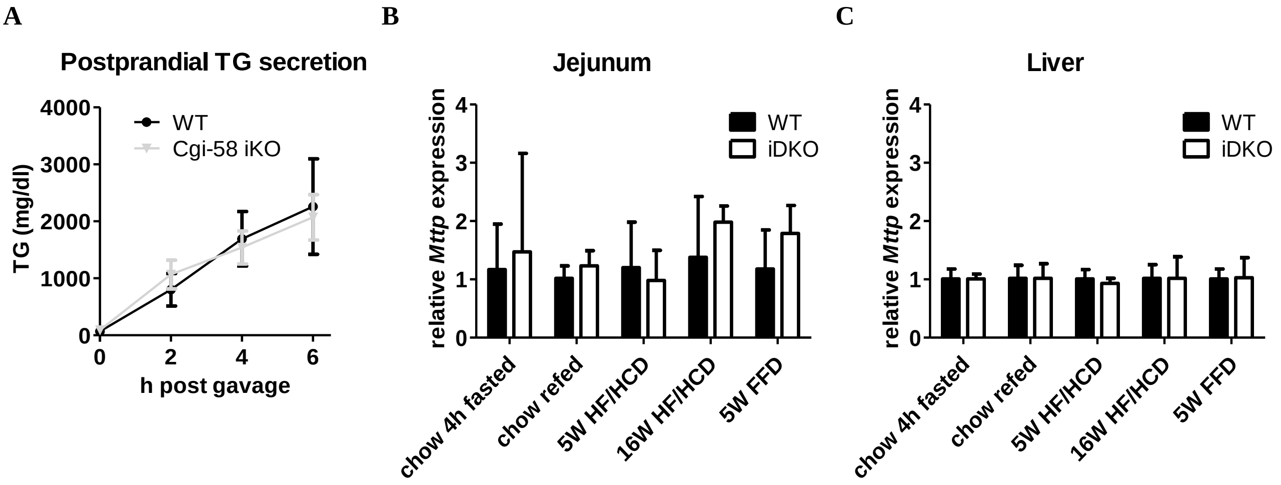

Figure S3: Intestinal ATGL/CGI-58 deficiency does not affect intestinal lipoprotein metabolism, related to Figure 3. A) Postprandial TG secretion in Cgi-58 iKO mice (n=5) after tyloxapol injection followed by an oral oil bolus (500 µl olive oil). B) Jejunal and C) hepatic *Mttp* mRNA expression in WT and iDKO mice. Data were normalized to the expression of cyclophilin A as housekeeping gene and represent mean (n=4-7) ± SD. *Mttp*, microsomal triglyceride transfer protein; HF/HCD, high-fat/high-cholesterol diet; FFD, fat-free diet.

Figure S4

A

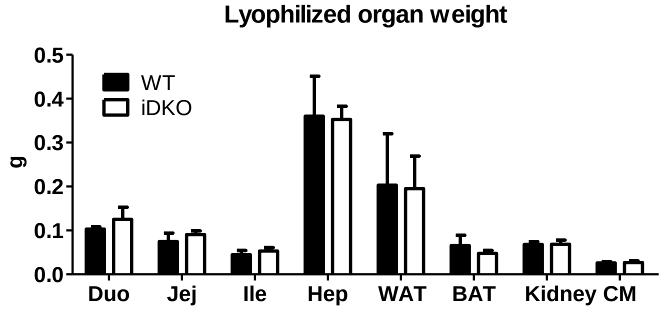

B

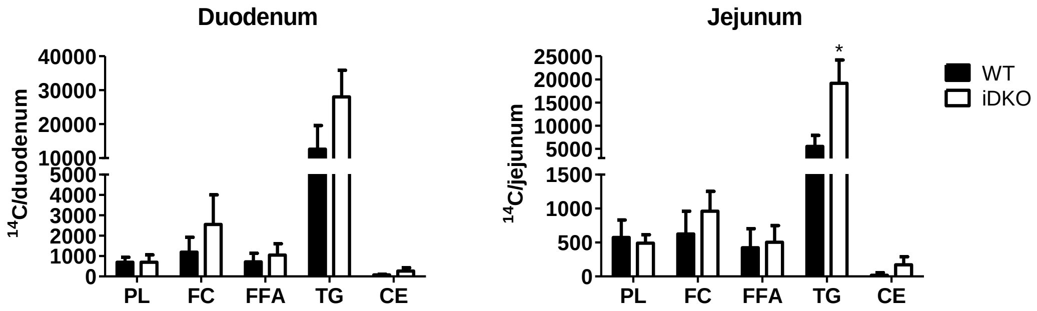

C

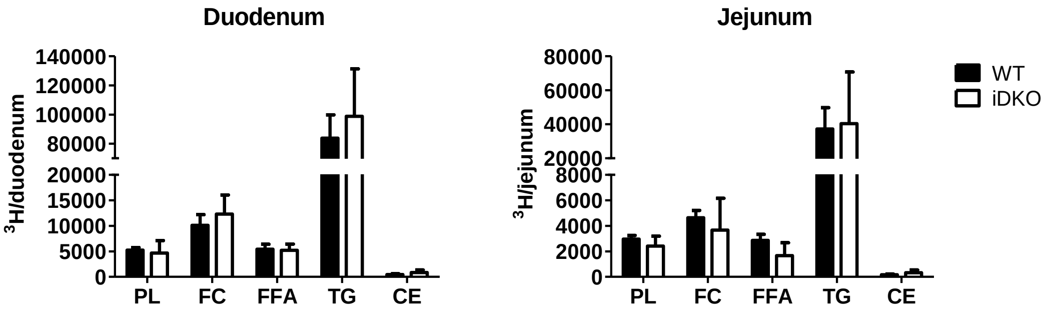

Figure S4: Lipids ingested with the first meal accumulate in the SI of iDKO mice mainly in the TG fraction, related to Figure 3. A) Lyophilized organ weights of WT and iDKO mice 10 h after administration of the first meal. B, C) After separation by thin-layer chromatography, radioactivity was determined in various lipid classes in the duodenum and jejunum of WT and iDKO mice. A) Distribution of ^{14}C-triolein, representing the first oil bolus. B) ^{3}H-triolein distribution, representing the second oil bolus. Data represent mean (n=3) + SD. * $p < 0.05$. Duo, duodenum; Jej, jejunum; Ile, ileum; Hep, hepar (liver) WAT/BAT, white/brown adipose tissue; CM, cardiac muscle (heart). PL, phospholipids; FC, free cholesterol; FFA, free fatty acids; TG, triglycerides; CE, cholesteryl esters.

Figure S5

A **Liver (30 min post i.v. injection)** **B** **Duodenum (30 min post i.v. injection)**

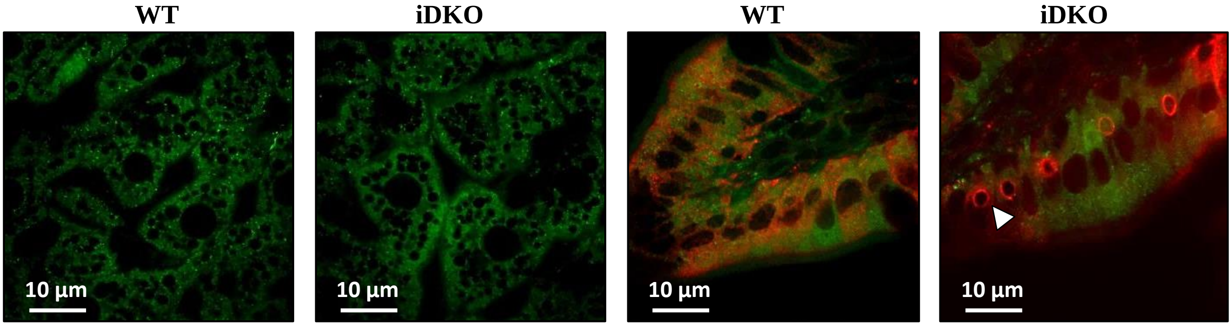

C

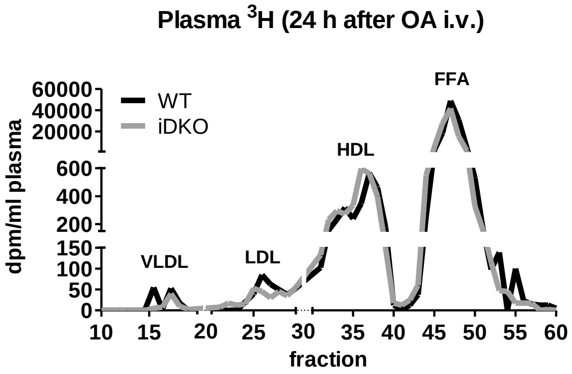

Figure S5: Distribution of intravenously applied FAs, related to Figure 4. Mice were injected i.v. with 20 µg BODIPY® C16 (green) in Intralipid and sacrificed 30 min post-injection. A) Hepatic and B) intestinal sections. Intestinal sections were counterstained with perilipin 3 (PLIN3, red) to visualize early cLD formation. Arrowheads indicate endogenous cLDs coated with PLIN3. Magnification, 100x; scale bar, 10 µm. C) Lipoprotein profile of 200 µl pooled plasma (n=6-7) 24 h after i.v. injection of 3H-OA. Before sacrifice, mice were fasted for 12 h. VLDL, very low-density lipoprotein; LDL, low-density lipoprotein; HDL, high-density lipoprotein; FFA, free fatty acid.

Figure S6

A **B**

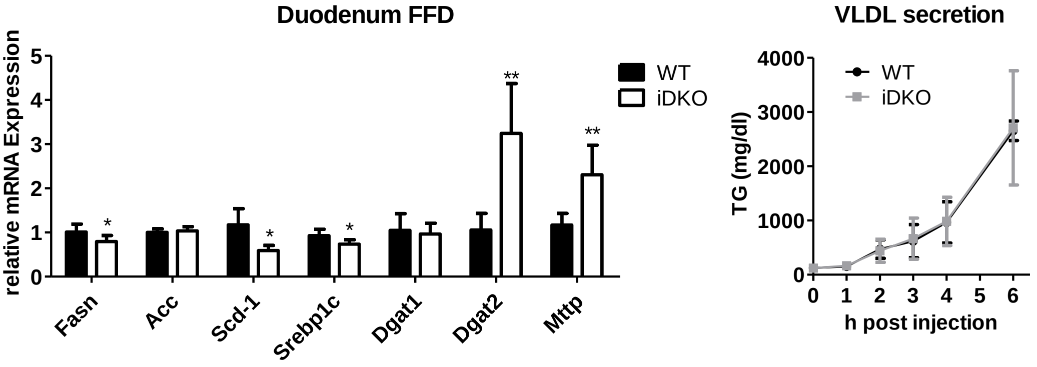

Figure S6: Atgl/Cgi-58 iDKO mice display decreased *de novo* lipogenesis, but increased *Dgat2* and *Mttp* mRNA expression after FFD feeding, related to Figure 5. A) qRT-PCR analysis of genes involved in *de novo* lipogenesis. Data (n=5-6) were normalized to cyclophilin A expression as housekeeping gene. B) Hepatic VLDL secretion in 16 h-fasted WT and iDKO mice (n=8). Mice were injected with tyloxapol (500 mg/kg bodyweight) prior to blood collection. Data represent mean ± SD. * $p < 0.05$; ** $p \leq 0.01$. *Fasn*, fatty acid synthase; *Acc*, acetyl-CoA carboxylase; *Scd-1*, stearoyl-CoA desaturase-1; *Srebp1c*, sterol regulatory element-binding protein 1; *Dgat*, acyl-CoA:diacylglycerol-acyltransferase; *Mttp*, microsomal triglyceride transfer protein.

Supplemental Table

Table S1: Primer sequences, related to STAR methods

**Gene**	**Forward primer**	**Reverse primer**
**Acc**	GGACTTGGAGCAGAGAACCTTCG	CAAGCTGGTTGTTGGAGGTGTA
**Atgl**	GCCACTCACATCTACGGAGC	GACAGCCACGGATGGTGTTC
**Ces1d**	ATGGAGGTGGACTGGTGGTG	AGTGCAGCCACCTGGTCCAA
**Ces1e**	CCAGTGACAGGGCAAATAGTC	GTAGACAGGACCAGTCCATCATA
**Ces1f**	CAAGTGGCTGCTCTGCATTG	TCCAGGAATGAAGGCCACAC
**Ces2a**	GTGGACTGGTTGTAGGATCAGC	TTCTTCTGCACCCAGCGTAAG
**Ces2c**	CCTGTAGGACCACTGCGATT	GAGATACAGGCAGTCCTCAG
**Ces2e**	CTTGATCCTGCCTCCCATCT	GATAGCGACCACCACCACAT
**Ces2f**	GCCTACCATTATACCTGACTCCC	CACAGCAGGCATAAACCTGAA
**Ces2g**	AGGTCCAAGGCAGGCTCAT	GGCCCTCCATATTCATCGTAACA
**Cgi-58**	GGTTAAGTCTAGTGCAGC	AAGCTGTCTCACCACTTG
**CyclophilinA**	CCATCCAGCCATTCAGTCTT	TTCCAGGATTCATGTGCCAG
**Ddhd2**	GAGTCCAGAGCGAAATGTCATC	GGCGTCCATGTCTATGAGTTC
**Dgat1**	TCCGCCTCTGGGCATTC	GAATCGGCCCACAATCCA
**Dgat2**	AGTGGCAATGCTATCATCATCGT	TCTTCTGGACCCATCGGCCCCAGGA
**Fasn**	GAAGCCGAACACCTCTGTGCAGT	GCTCCTTGCTGCCATCTGTATTG
**Hsl**	GCTGGTGACACTCGCAGAAG	TGGCTGGTGTCTCTGTGTCC
**Lal**	CGGCTTGCTGGCAGATTCTA	GTGCAGCCTTGAGAATGACC
**Mgl**	CGGACTTCCAAGTTTTTGTCAGA	GCAGCCACTAGGATGGAGATG
**Mttp**	GTCAACAGAGAGGCGAGAAG	CTAGCCAAGCCTCTCTTGAG
**Ptl**	GCCATTGGAAGGATCACAGG	CCTGGCATTTCGATTCCTCC
**Scd-1**	CCGGAGACCCCTTAGATCGA	TAGCCTGTAAAAGATTTCTGCAAACC
**Srebp1c**	CCATCGACTACATCCGCTTCTT	ACTTCGCAGGGTCAGGTTCTC
